# A Tumor Microenvironment-Driven Network Regulated by STAT3 and p65 Negatively Controls the Enrichment of Cancer Stem Cells in Human HR+/HER2− Breast Cancer

**DOI:** 10.3390/cancers15082255

**Published:** 2023-04-12

**Authors:** Hagar Ben-Yaakov, Tsipi Meshel, Metsada Pasmanik-Chor, Cindy Körner, Adit Ben-Baruch

**Affiliations:** 1 The Shmunis School of Biomedicine and Cancer Research, George S. Wise Faculty of Life Sciences, Tel Aviv University, Tel Aviv 6997801, Israel; 2 Bioinformatics Unit, George S. Wise Faculty of Life Sciences, Tel Aviv University, Tel Aviv 6997801, Israel; 3 Division of Molecular Genome Analysis, German Cancer Research Center, 69120 Heidelberg, Germany

**Keywords:** cancer stem cells, epidermal growth factor, estrogen, HR+/HER2− breast cancer, p65, STAT3, tumor microenvironment, tumor necrosis factor α

## Abstract

**Simple Summary:**

Hormone receptor-positive (HR+)/HER2− breast cancer is driven by extracellular cues within the tumor microenvironment (TME) including hormonal, inflammatory and growth-stimulating signals. Our past findings indicate that a “TME Stimulation” jointly addressing these three arms induces pro-metastatic traits in HR+/HER2− breast cancer cells, primarily with the enrichment of cancer stem cells (CSCs), driving metastasis in vivo. Here, we reveal intricate roles for STAT3 as a negative and positive regulator of TME Stimulation-induced pro-metastatic effects in HR+/HER2− cells. Of note, the two transcription factors STAT3 and p65 acted in cooperativity to limit CSC enrichment, and their down-regulation has led to enriched levels of chemotherapy-resistant CSCs. Moreover, STAT3 and p65 activation were inversely connected to a CSC signature and positively associated with improved survival in patient datasets. These findings suggest that we need to carefully consider the roles of STAT3 and p65 roles in regulating TME activities in malignant diseases, in efforts to identify novel targets for therapeutic intervention.

**Abstract:**

Hormone receptor-positive and HER2-negative (HR+/HER2−; luminal A) tumors are prevalent in breast cancer. Our past studies demonstrated that “TME Stimulation” (estrogen + TNFα + EGF, representing three arms of the tumor microenvironment, TME) has enriched metastasis-forming cancer stem cells (CSCs) in HR+/HER2− human breast cancer cells. Here, following information obtained by RNAseq analyses of TME-stimulated CSCs and Non-CSCs, we found that TME Stimulation has induced the activation of S727-STAT3, Y705-STAT3, STAT1 and p65. Upon TME Stimulation, stattic (STAT3 inhibitor) usage demonstrated that Y705-STAT3 activation negatively controlled CSC enrichment and epithelial-to-mesenchymal transition (EMT) traits, while inducing CXCL8 (IL-8) and PD-L1 expression. However, STAT3 knock-down (siSTAT3) had no effect on these functions; in terms of CSC enrichment, p65 had down-regulatory roles that compensated for the loss of an entire STAT3 protein. Y705-STAT3 and p65 acted additively in reducing CSC enrichment, and Y705A-STAT3 variant + sip65 has enriched chemo-resistant CSCs. Clinical data analyses revealed an inverse correlation between Y705-STAT3 + p65 phosphorylation and CSC signature in luminal A patients, and connection to improved disease course. Overall, we find regulatory roles for Y705-STAT3 and p65 in TME-stimulated HR+/HER2− tumors, with the ability to limit CSC enrichment. These findings raise concerns about using inhibitors of STAT3 and p65 as therapeutic strategies in the clinic.

## 1. Introduction

A large proportion of breast cancers express receptors for estrogen/progesterone (hormone receptor-positive, HR+) while lacking the over-expression of HER2 (HER2−) (corresponding mainly to the luminal A PAM50 categorization). The prognosis of HR+/HER2− tumors is the best out of all breast cancer subtypes, yet in many of the patients the tumors eventually develop resistance to therapy and cancer cells metastasize, leading to reduced patient survival [1,2,3,4].

The transition of HR+/HER2− breast cancer cells to a more aggressive phenotype (e.g., HR− phenotype) may stand on the basis of poor prognosis of some of the patients. Several studies demonstrated transitions of primary HR+ breast tumors to an HR− status in metastases in the same patients, and in tumors recurring after treatment; such a shift was connected to reduced survival [5,6]. Additionally, luminal characteristics have been suggested for the aggressive “luminal androgen receptor” (LAR) subtype of triple-negative breast tumors (TNBC) [7]. In a mouse model system, the transition of luminal mammary cancer cells to highly metastatic claudin-low cells was reported [8].

Many mechanisms can contribute to the transition of HR+/luminal A breast tumor cells towards a more advanced phenotype, with roles attributed to progenitor and cancer stem cells (CSCs) and to increased stemness in this process; in this context, recent studies have shown that mammary CSCs of luminal origin can differentiate in vivo to the highly aggressive subtypes TNBC, claudin-low and HER2+ [9,10,11]. Moreover, luminal progenitor cells were found to be sensitive to BRCA1 mutations and prone to acquire basal-like properties [11].

CSCs have been identified in breast cancer by different markers (primarily being CD44^+^/CD24^low/−^), and by their ability to generate a heterogenous cell population, resist chemotherapy and express an epithelial-to-mesenchymal (EMT) phenotype [12,13,14]. The potential roles of CSCs in the transition of luminal breast cancer cells to a highly aggressive phenotype emphasize the need to identify the mechanisms that increase CSC proportions in HR+/HER2− breast cancer cells. The generation and functions of CSCs in breast cancer were found to be regulated by intrinsic molecular mechanisms (e.g., p53, slug, NOTCH), as well as by tumor microenvironment (TME)-driven events, with the involvement of factors belonging to the Wnt pathway, hepatocyte growth factor, transforming growth factor β and others [11,15].

Within the TME, we have previously identified the ability of joint stimulation by estrogen, tumor necrosis factor α (TNFα) and epithelial growth factor (EGF) to profoundly elevate the content of CSCs in HR+/HER2− breast cancer cells [16]. Our studies of this combined stimulation have addressed the joint effects of factors of the hormonal, inflammatory and growth-promoting arms to act together and influence the pro-metastatic functions of HR+/HER2− breast cancer cells. In this setting, estrogen was chosen in view of its being the hormone with most robust abilities to promote the aggressiveness of HR+/HER2− breast cancer cells [17,18]. In parallel, TNFα was used as a representative of cancer inflammation, a process considered as prime inducer of cancer progression [19,20,21,22]. TNFα is a most potent pro-inflammatory cytokine whose presence was associated with breast cancer progression, and possibly due to its chronic expression at the tumor sites, it is strongly connected to advanced malignancy in patients and in animal breast models [21,22,23,24,25,26,27]. Additionally, EGF represented the arm of growth factors that can elevate pro-malignancy activities in HR+/HER2− breast cancer cells, acting primarily via EGFR [28,29,30,31,32,33].

In our published studies we have demonstrated that “TME Stimulation” combining the three elements together was more effective than each factor alone in promoting tumor-supporting phenotypes in HR+/HER2− breast cancer cells [34]. Among others, TME Stimulation has given rise to increased bone metastasis of HR+/HER2− breast cancer cells and enriched the presence of CSCs in the cell population [16,35]. These cells were characterized as CSCs based on their ability to regenerate the entire cell population, to express EMT-related phenotypes, to better resist chemotherapy and to drive metastasis in vivo, in mice studies [16].

To follow up on these observations, in the current research we investigated further the impact of TME Stimulation on pro-metastatic characteristics and functions of HR+/HER2− breast tumor cells, focusing mainly on the mechanisms accounting for CSC enrichment. Our data demonstrate that upon TME Stimulation, STAT3 had opposing roles in regulating pro-tumor activities in HR+/HER2− breast cancer cells. By using stattic, which inhibited the phosphorylation of STAT3 at the Y705 position, we found that Y705-STAT3 negatively regulated tumor cell intrinsic pro-metastatic functions of CSC enrichment and EMT, while inducing TME-related pro-metastatic functions, namely the release of CXCL8 (IL-8)—which is a strong inflammatory chemokine playing causative roles in promoting breast cancer progression, partly through the recruitment of deleterious neutrophils to tumors [36,37]—and expression of the inhibitory immune checkpoint PD-L1.

Furthermore, through activation kinetics and inhibition of specific transcription factors, we revealed an intricate network that regulated CSC enrichment in these cells, comprising cross-regulatory functions between STAT3 and p65. Here, we found that the loss of the entire STAT3 protein (by siRNA to STAT3) was compensated by p65 activities (NF-κB pathway), that recapitulated for STAT3 absence and down-regulated CSC enrichment. The dual down-regulation of Y705-STAT3 and p65 activities has led to further increased presence of CSCs in TME-stimulated cells, demonstrating additive activities of the two transcription factors. Moreover, doxorubicin resistance studies and clinical information from the TCGA patient dataset have demonstrated the connection of activated Y705-STAT3 + p65 to the clinical setting, including their inverse relation with the CSC gene signature and their association with good prognosis, in luminal A patients.

Thus, our study indicates that under conditions of HR+/HER2− cell exposure to combined stimulation by TME factors—in this specific case estrogen, TNFα and EGF as representatives of different arms of the tumor milieu—STAT3 and p65 have combined non-conventional roles as negative regulators of CSC enrichment. In light of their well-described pro-metastatic roles in other aspects of malignancy in breast cancer [38,39,40,41,42,43], these findings call for the careful consideration of the therapeutic effects of inhibitors targeting STAT3 and p65 in the clinic.

## 2. Materials and Methods

### 2.1. Cell Cultures

Human MCF-7 (ATCC) and T47D cells (provided by the researcher who generated this cell line, Prof. Keydar at Tel Aviv University, Tel Aviv, Israel) were cultured in growth medium containing DMEM (4.5 g/L glucose) supplemented by 10% fetal bovine serum (FBS), 2% L-glutamine and 1% penicillin-streptomycin-amphotericin solution, termed herein “complete media” (all materials were from Biological Industries, Beit Ha’emek, Israel or from Sigma, Saint Louis, MO, USA).

### 2.2. Cell Exposure to TME Stimulation

MCF-7 and T47D cells were plated overnight in complete media, washed in PBS, and stimulated with TNFα + estrogen + EGF (termed herein “TME Stimulation”) in phenol red-free DMEM containing 1% dialyzed serum (Biological Industries), for time points indicated in the Figure legend. The concentrations of exposure to the three stimulants were selected based on titration and kinetics analyses; concentrations agree with the conventional dose range used in other research systems [31,44,45]: TNFα: 50 ng/mL (#300-01A; PeproTech, Rocky Hill, NJ, USA); estrogen: 10^−8^ M (#E8875; Sigma); EGF: 30 ng/mL (#236-EG; R&D systems, Minneapolis, MN, USA). In all procedures, control non-stimulated cells were grown in the presence of the diluents of the above stimulators (For TNFα: 0.1% BSA in DDW; for estrogen: ethanol; for EGF: 0.1% BSA in DDW + 10 mM acetic acid).

When indicated, the following pharmacological inhibitors were used at conventional concentrations: Stattic: 5 μM (#S7947; Sigma); Bay 11-7082: 5 μM (Bay, #B5556; Sigma). The inhibitors were added to cell cultures two h prior to the stimulation of the cells by TNFα + estrogen + EGF or to control vehicle-treated cells and were present in culture throughout the duration of stimulation (as indicated in Figure legends). The inhibitors did not have a marked inhibitory effect on cell growth (it was even increased to some extent with stattic). When relevant, cells were exposed to 0.5 μM doxorubicin (#44583; Sigma) during TME Stimulation; doxorubicin concentration was selected based on titration analyses (0.5 μM doxorubicin has led to 57 ± 5% cell death in TME-stimulated cells, based on cell count with exclusion dye (trypan blue). Control cells were treated with the vehicle of the drugs at similar dilutions (DMSO; Sigma).

### 2.3. Flow Cytometry Analyses

Expression levels of cell surface molecules were determined by flow cytometry in viable TME-stimulated and vehicle-treated MCF-7 and T47D cells, using a CytoFlex LX flow cytometer (Beckman Coulter, Indianapolis, IN, USA). The following antibodies were used: Alexa 488-conjugated rat IgG2b against CD44 (#103016; Biolegend, San Diego, CA, USA); APC-conjugated rat IgG2b against CD44 (#103011; Biolegend); PE-conjugated mouse IgG2a against CD24 (#560991; BD Biosciences, Franklin Lakes, NJ, USA); PE-conjugated mouse IgG1 against PD-L1 (CD274) (#12-5983-42; eBioscience, San Diego, CA, USA); APC-conjugated mouse IgG1 against E-cadherin (#324107; Biolegend). Baseline staining was obtained by non-relevant isotype-matched controls. Staining patterns were determined using the Flowjo v10 software (BD Biosciences).

### 2.4. ELISA Analyses

TME-stimulated and vehicle-treated MCF-7 and T47D cells were grown (as described above) for different time points (as described in Figure legends). Then, conditioned media (CM) were removed and CXCL8, IL-6, IFNβ or IFNγ levels were determined by ELISA using standard curves at the linear range of absorbance. To this end, recombinant human CXCL8 (rhCXCL8; #200-08; PeproTech) or rhIFNβ (#300-O2BC; Peprotech) were used. Coating polyclonal antibodies (CXCL8: #500-P28, IFNβ: #500-P32B; PeproTech) and detecting biotinylated rabbit polyclonal antibodies (CXCL8: #500-P28Bt, IFNβ: #500-P32BBT; PeproTech) were used. After the addition of streptavidin-horseradish peroxidase (#016-030-084; Jackson Immunoresearch Laboratories, West Grove, PA, USA), substrate TMB/E solution (#ES001; Millipore, Temecula, CA, USA) was added. For IL-6 and IFNγ, the following ELISA kits were used: IL-6 (#900-TM16; Peprotech), IFNγ (#900-TM27, Peprotech). The reaction was stopped by the addition of 0.18 M H_2_SO_4_ and was measured at 450 nm.

### 2.5. Transcriptome RNAseq Analyses

To determine transcriptome alterations, genome-wide expression analysis was performed by RNAseq experiments. MCF-7 cells were exposed to TME-stimulation or vehicles for 96 h as described above. Then, cells were stained by fluorescently-labeled antibodies against CD44 and CD24 (as described above); CD44^+^/CD24^low/−^ cells were sorted as CSCs, and the rest of the population was collected as Non-CSCs. Non-stimulated cells were also passed through the sorter to compare conditions. Cells were sorted using a Becton Dickinson FACSAria (BD Biosciences). RNA was extracted from vehicle-treated cells, TME-stimulated CSCs and TME-stimulated Non-CSCs in three independent biological repeats. Total RNA was isolated using RNA extraction kit RNeasy Micro Kit (#74004, Qiagen, Hilden, Germany).

RNA libraries were prepared using KAPA Stranded RNA-seq Kit with RiboErase (HMR) (#KR1151; Roche, Basel, Switzerland), then submitted to analysis using NextSeq 500/550 High Output Kit v2.5 (75 Cycles) (#20024906; Illumina, San Diego, CA, USA) for RNAseq, at the Genomics Unit of Tel Aviv University. Samples were sequenced on Illumina Nextseq500 (Illumina). The output was >20 million reads in most samples. Trimming was performed on low-quality reads.

The processed reads were mapped to human genome, hg38, using STAR v2.7.3a [46] and then the number of reads that were mapped to each gene was calculated using Partek E/M algorithm. Data of these transcriptome analyses were deposited in NCBI’s Gene Expression Omnibus [47] and are accessible through GEO Series accession number GSE226339. Statistical analyses were performed by DESeq2 statistical test at cutoff of fold change (FC) ≥ 2 or FC ≤ −2, with pFDR < 0.05. Heatmaps were generated with Partek Genomics Suite (Partek Genomics Suite^®^; version 7.19.1125), using hierarchical clustering with Pearson’s correlation distance dissimilarity measures and Ward’s method. The functional enrichment analysis was performed using g:Profiler [48] (version e107_eg54_p17_bf42210) with g:SCS multiple testing correction method applying significance threshold of 0.05.

### 2.6. Western Blot Analyses

MCF-7 cells have been TME-stimulated or treated by vehicles (as described above) for different time points (as described in Figure legends). Following lysis in RIPA buffer, Western blot (WB) was performed using antibodies from Cell Signaling Technology (CST, Danvers, MA, USA), except where otherwise indicated: Total (T)-STAT3: (#4904); Phosphorylated (P)-STAT3 Y705: (#9145); (P)-STAT3 S727: #9134; (T)-STAT1: #9172; (P)-STAT1: #9167; (T)-p65: #8242; (P)-p65: #3033. GAPDH (#ab9485; Abcam, Cambridge, UK) or β-Tubulin (#2146) served as loading controls. Then, the membranes were incubated with streptavidin-horseradish peroxidase (HRP)-conjugated goat anti-rabbit IgG (#111-035-003; Jackson ImmunoResearch Laboratories). The membranes were subjected to enhanced chemiluminescence (ECL) analysis (#WBLUR0500, Merck, Darmstadt, Germany), and were visualized with Amersham ImageQuant 800 (GE Healthcare, Little Chalfont, UK). Quantification was carried out using ImageJ software (version 1.4).

### 2.7. Knock-Down of Target Genes

Knock-down (KD) of STAT3, STAT1 and/or p65 (RELA) by transient siRNA transfections was performed in MCF-7 and T47D cells (as appropriate) using the Lipofectamine RNAiMAX transfection reagent (#13778075; Invitrogen, Grand Island, NY, USA) according to manufacturer’s instructions (following titration analyses), in a reverse transfection protocol. The following ON-TARGET plus siRNA SMART pools were used (10 nM; all from Dharmacon, Lafayette, CO, USA): STAT3 siRNA (siSTAT3): #L-002000-00; STAT1 siRNA (siSTAT1): #L-003543-00; p65 siRNA (sip65): #L-003533-00. siRNA control (siCTRL) was introduced by ON-TARGET plus non-targeting control siRNA pool (#D-001810-10). 72 h after transfection, cells were stimulated as described above and used in assays, as necessary. KD of target genes was validated by WB analyses. In general, siRNA expression did not affect cell viability (except for a reduction noted when siRNAs to STAT3, STAT1 and p65 were expressed together).

### 2.8. Generation of Cells Over-Expressing Wild Type STAT3 or Mutant STAT3

To generate wild type (WT) and mutant Y705A-STAT3 constructs, total RNA was extracted from MCF-7 cells and RT reaction was performed. The construct of human WT-STAT3 variant 1 (NM_139276.2) was created by PCR amplification of the above cDNA, using the STAT3(188)-Age1-sense primer AAAGCGACCGGTATGGCCCAATGGAATCAGCTACA and the STAT3(2500)-Pac1-anti-sense primer ATACTATTAATTAATCACATGGGGGAGGTAGCGC. To generate Y705A-STAT3, we used sense primer STAT3(Y705A-2341) GCGCTGCCCCAGCCCTGAAGACCAAG and anti-sense primer STAT3(Y705A-2367) CTTGGTCTTCAGGGCTGGGGCAGCGC. The generated fragments were digested with Age1 and Pac1 and ligated into the corresponding sites of pQCXIP vector (https://www.addgene.org/vector-database/3870/, accessed on 5 December 2022); Clontech, Santa Clara, CA, USA). PCR products of STAT3 were sequenced and found to be identical to the published sequence (except for the mutation site).

Then, HEK-293 cells were co-transfected by calcium chloride with 10 μg of each of the vectors, 7.5 μg helper plasmid encoding gag-pol and 2.5 μg helper plasmid encoding vesicular stomatitis virus-G (VSV-G) proteins. Supernatants were collected after 48 h, filtered through a 0.45 μm mesh, and incubated with MCF-7 cells in the presence of 8 mg/mL polybrene for 6 h. Then, 72 h following infection, the cells underwent selection with 1 μg/mL puromycin (#P-1033; AG Scientific, San Diego, CA, USA) for 48 h.

MCF-7 cells were infected with either WT-STAT3 or Y705A-STAT3 pQCXIP vectors (or with sham PQCXIP vector as control) and STAT3 expression and phosphorylation were validated by WB analyses. The growth rate of WT-STAT3-infected cells was similar to that of cells expressing Y705A-STAT3.

### 2.9. Analyses of Patient Databases

mRNA data from The Cancer Genomic Atlas (TCGA) breast cancer (BRCA) database had been aligned to the GRCH38 build (hg38) in the course of the GDC harmonizing efforts [49,50]. They were downloaded from the GDC harmonized database using the Bioconductor R package TCGA biolinks (version 2.12.6). Clinical survival and recurrence parameters were obtained from Liu et al. [51]. Read counts from the htseq-count analysis were provided for 1222 BRCA patients and 60,483 genes. We excluded from the analysis 16 mitochondrial (MT)-genes (MT-CO3, MT-CO2, MT-CO1, MT-ND4, MT-RNR2, MT-ATP6, MT-CYB, MT-ND1, MT-ND3, MT-ND2, MT-ATP8, MT-ND4L, MT-ND6, MT-ND5, MT-RNR1 and MT-TP), because they introduced substantial bias for a subset of patients due to extremely high expression values. Samples with a tumor purity lower than 40% were excluded to improve data quality. Read counts for the remaining 1153 samples were converted to transcripts per million (tpm), and data from luminal A patients with available reverse phase protein array (RPPA) data were filtered for inclusion in the analyses (308 samples). After this selection process, only those genes having HGNC symbols were analyzed.

Gene set enrichment analysis (GSEA) was performed on mRNA data obtained from luminal A breast cancer patients from the TCGA dataset using the GSEA software (version 4.2.3) [52]. As the gene set, the list of genes determined to be significantly down-regulated in CSCs vs. Non-CSCs (FC < −2, pFDR < 0.05, *n* = 279, as described above; Table S1) was used. Phosphoproteomics values were obtained using UCSC Xena platform [53]. The TCGA Breast Cancer (BRCA) study was selected and filtered based on sample type and PAM50 subtypes to contain only samples from primary tumors classified as luminal A (“LumA”). Phosphoproteomics values based on RPPA were then extracted for the following proteins: P-Y705-STAT3 (STAT3_pY705-R-V) and P-p65 (NF-kB-p65_pS536-R-C). Data on P-S727-STAT3 was not available in the database. GSEA was performed using a Spearman Correlation between mRNA expression and P-Y705-STAT3, P-p65 or the sum of P-Y705-STAT3 + P-p65 values from the TCGA RPPA analyses as the weighted ranking metric. Statistical significance was assessed by 1000 permutations by phenotype.

Survival analyses based on abundance of P-Y705-STAT3 + P-p65 and progression-free interval (PFI) were performed using a Kaplan–Meier Analysis and Log-Rank Mantel-Cox test in GraphPad Prism (version 9.0); progression within the first three years of follow-up was considered an event, and patients without progression after three years were considered as censored to investigate the three-year PFI. In analyses, the upper quartile of expression of “High P-Y705-STAT3 + High P-p65” (*n* = 72) was compared to the rest of the patients, defined as “Low in any” (*n* = 236).

### 2.10. Data Presentation and Statistical Analyses

All experiments were performed in *n* ≥ 3 independent experimental repeats demonstrating similar results; generally, a representative experiment out of these independent repeats is presented in the figures. Statistical analyses of transcriptome and TCGA studies were described in their relevant sections. Statistical analyses of all other assays were performed by two-tailed unpaired or paired Student’s *t*-tests. *p* < 0.05 was considered statistically significant.

## 3. Results

### 3.1. TME Stimulation Promotes Multiple Tumor-Supporting Characteristics and Functions in HR+/HER2− Breast Cancer Cells

In our previous studies analyzing the impact of TME Stimulation on HR+/HER2− cells, we have identified an increase in the proportions of CSCs in cultures of MCF-7 and T47D cells following 72 h of TME Stimulation [16]. Under similar conditions of 72 h of TME Stimulation, MCF-7 cells have also demonstrated increased EMT-related properties (determined by the reduced expression of E-cadherin and elevated expression of zeb1, snail and slug) and elevated levels of CXCL8 (IL-8) [16,34]. Jointly, these TME Stimulation-induced alterations, and particularly CSC enrichment, have led to increased metastatic potential of the cells, in vivo.

To enable the determination of the molecular mechanisms mediating the effects of TME Stimulation on HR+/HER2− breast cancer cells, we were looking for conditions that would potentiate the effects of TME Stimulation and will enable us to detect additional pro-metastatic phenotypes in the cells. To this end, in current research we extended TME Stimulation of both MCF-7 and T47D cells to 96 h.

**Figure 1:**
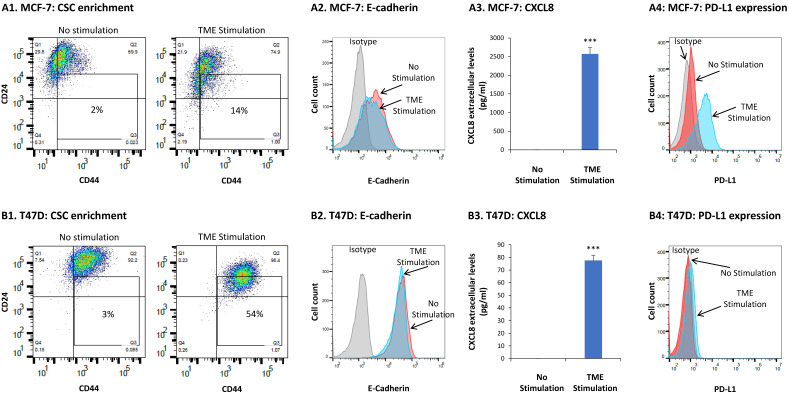
TME Stimulation leads to intrinsic and TME-related pro-metastatic effects in MCF-7 and T47D HR+/HER2− breast cancer cells: MCF-7 (**A**) and T47D (**B**) HR+/HER2− human breast tumor cells were exposed to TME Stimulation in culture (estrogen 10^−8^ M, TNFα 50 ng/mL, EGF 30 ng/mL) or to vehicles only (“No stimulation”) for 96 h. The stimulatory conditions were selected following titration and kinetics analyses. Concentrations agree with the conventional dose range used in other research systems [31,44,45]. (**A1**,**B1**) The contents of CSCs, determined as CD44^+^/CD24^low/−^ cells. Cell surface CD44 and CD24 expression was determined by flow cytometry, using fluorescently-labeled specific antibodies. Isotype-matched control antibodies were used to determine baseline staining (not shown). (**A2**,**B2**) EMT-related properties, manifested by cell surface expression of E-cadherin, were determined by flow cytometry using fluorescently-labeled specific antibodies. Isotype-matched control antibodies were used to determine baseline staining (Isotype). (**A3**,**B3**) CXCL8 expression was determined in the conditioned media (CM) of the cells by ELISA, at the linear range of absorbance. (**A4**,**B4**) Cell surface PD-L1 expression was determined by flow cytometry using fluorescently-labeled specific antibodies. Isotype-matched control antibodies were used to determine baseline staining (Isotype). In all panels, the results are from a representative experiment of *n* ≥ 3, showing similar results. *** *p* < 0.005 in MCF-7 cells and *p* < 0.001 in T47D cells.

Under these conditions, TME Stimulation of MCF-7 cells has led to an increase in the proportions of CSCs—identified as CD44^+^/CD24^low/−^ cells—from 2.3 ± 0.9% in vehicle-treated cells to 15.4 ± 5.6% in TME-stimulated cells (a representative experiment of *n* ≥ 3 is demonstrated in Figure 1A1). Additionally, we noticed that TME Stimulation has induced a small shift in MCF-7 cells towards an EMT-like phenotype, as indicated by reduced expression levels of E-cadherin (Figure 1A2).

In parallel to these two intrinsic pro-metastatic phenotypes of cancer cells, we analyzed the effects of TME Stimulation on tumor cell functions that can affect the inflammatory and immune contexts/activities of the TME. First, we determined the impact of TME Stimulation on the release of CXCL8. Here, we found a very strong induction of CXCL8 following the exposure of MCF-7 cells to TME Stimulation (Figure 1A3). Next, we addressed the effects of TME Stimulation on the expression of PD-L1, a major inhibitory immune checkpoint that leads to reduced anti-tumor immune activities in cancer [54]. The findings of Figure 1A4 demonstrate a prominent increase in PD-L1 cell surface expression, which was induced in MCF-7 cells by TME Stimulation.

Similar analyses performed on T47D cells revealed a strong impact of TME Stimulation on two of the above parameters. A very potent CSC enrichment was noted in T47D cells, where CSCs levels were elevated from 2.1 ± 0.9% in vehicle-treated cells to 40.5 ± 18% in TME-stimulated cells (Figure 1B1). A marked increase in CXCL8 expression was also found in T47D cells exposed to TME Stimulation (Figure 1B3). The other two parameters, EMT and PD-L1 expression were not affected or only minimally increased by TME Stimulation of T47D cells (Figure 1B2,B4, respectively).

Overall, the above findings indicate that combined stimulation of HR+/HER2− human breast cancer cells (MCF-7 and T47D) by factors that represent three different arms of the TME—hormonal, inflammatory and growth-promoting—strongly promotes the proportions of CSCs, the release of the pro-metastatic chemokine CXCL8 and PD-L1 expression (the latter by MCF-7 cells only).

### 3.2. TME Stimulation Leads to Pronounced Alterations in Gene Signatures of HR+/HER2− Breast Cancer Cells

The findings of Figure 1 indicate that the HR+/HER2− cell population contains a low proportion of CSCs, whose levels were markedly promoted by TME Stimulation. These CSCs co-exist in the tumors in proximity to Non-CSC cells, suggesting that interactions between the two cell subsets may take place. To decipher genes whose expression may distinguish vehicle-treated HR+/HER2− cells from TME-stimulated CSC and TME-stimulated Non-CSC subsets, and to identify transcriptome characteristics of the CSC and Non-CSC sub-populations, RNAseq studies were performed on MCF-7 cells.

**Figure 2:**
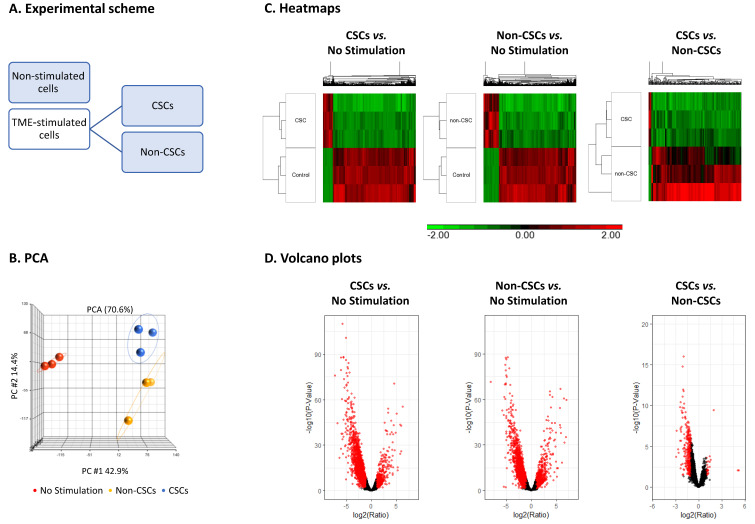
TME Stimulation leads to substantial modifications in gene expression in TME-stimulated CSCs and TME-stimulated Non-CSCs in MCF-7 HR+/HER2− cells: MCF-7 cells that were exposed to TME Stimulation for 96 h (concentrations as described in Figure 1) were sorted based on the CD44^+^ and CD24^low/−^ markers to CSCs and Non-CSCs. In parallel, a group of non-stimulated cells (“No Stimulation”; includes the entire cell population) was analyzed (after 96 h in culture with the vehicles). Gene expression in these three groups of cells were determined by RNAseq. Cell sorting and RNAseq analyses were performed on three independent biological repeats. (**A**) An experimental scheme of the experiment, noting in blue the cell groups that were analyzed by RNAseq. (**B**) Principal component analysis (PCA) of three replicates in each of the three cell groups. (**C**) Heatmaps and (**D**) volcano plots of differentially expressed genes that passed the cutoff FC ≥ 2 or FC ≤ −2, with pFDR < 0.05 for the following comparisons: TME-stimulated “CSCs” compared to “No Stimulation”; TME-stimulated “Non-CSCs” compared to “No Stimulation”; TME-stimulated “CSCs” compared to TME-stimulated “Non-CSCs”. In Part (**D**), red dots mark genes passing the FC and significance criteria.

Here, three independent experiments were performed, each containing the following three cell subsets (marked in blue, Figure 2A): (1) non-stimulated cells, treated by the vehicles of TME Stimulation; because these cells hardly contain any CSCs (2.3 ± 0.9%, Figure 1A1), in this case, the entire cell population was analyzed, as a whole. (2) TME-stimulated CSCs, characterized as CD44^+^/CD24^low/−^ cells; (3) TME-stimulated Non-CSCs. These cells were those remaining following sorting of the CSCs from TME-stimulated cell cultures (based on the CD44/CD24 markers).

The transcriptomes of these three cell populations were analyzed by RNAseq studies. The PCA analyses in Figure 2B reveal a clear distinction between the three analyzed cell groups. Figure 2C,D demonstrate heatmaps and volcano plots comparing between the different cell sub-populations, based on a cutoff of fold change (FC) > 2 or FC < −2, and pFDR < 0.05. The results of Figure 2C demonstrate marked differences in transcriptome between TME Stimulation-derived CSCs and non-stimulated cells (additional information is provided in Table S2). Major alterations in gene expression were also noted between TME Stimulation-derived Non-CSCs and non-stimulated cells (Figure 2C and Table S3). TME-stimulated CSCs demonstrated mostly down-regulated genes, when they were compared to TME-stimulated Non-CSCs (Figure 2C and Table S4). Figure 2D shows that the majority of genes differing between TME-stimulated CSCs and TME-stimulated Non-CSCs are not as many as in their comparisons to non-stimulated cells and are mostly down-regulated in the CSCs compared to Non-CSCs (genes passing the FC and significance criteria are presented in red, in Figure 2D).

**Figure 3:**
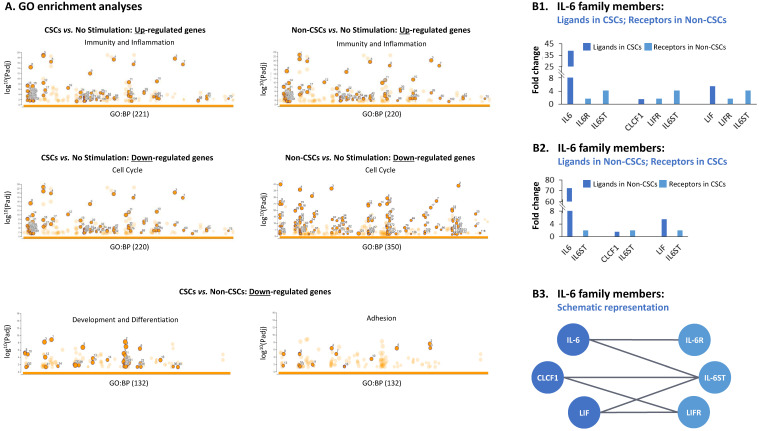
Differences in gene expression induced by TME Stimulation in CSCs and Non-CSCs, and expression profiles of IL-6 family members in HR+/HER2− breast cancer cells: The Figure demonstrates analyses of the gene expression data of the three MCF-7 cell groups described in Figure 2 (TME-stimulated “CSCs”, TME-stimulated “Non-CSCs”, “No Stimulation”) using differentially expressed genes that passed the cutoff FC ≥ 2 or FC ≤ −2 with pFDR < 0.05. (**A**) Enrichment analyses for GO terms (biological processes) were performed, using g:Profiler tool. The Figure demonstrates Manhattan plots for all enriched GO terms for the following gene lists: upper panel—up-regulated genes in TME-stimulated “CSCs” and TME-stimulated "Non-CSCs", each compared to “No Stimulation”; center panel—down-regulated genes in TME-stimulated “CSCs” and TME-stimulated “Non-CSCs”, each compared to “No Stimulation”; lower panel—down-regulated genes in TME-stimulated “CSCs” compared to TME-stimulated “Non-CSCs”. In all panels, circles represent GO terms enriched for the relevant gene list. The circles highlighted by frame represent the GO terms relevant to the sub-titled process. Numbers in the X axis denote the number of pathways enriched in each comparison. Information on all pathways demonstrated in these analyses is provided in Table S5. (**B**) Among all differentially expressed genes that passed the cutoffs in RNAseq analyses, cytokines and chemokines and their receptors were filtered. Among these differentially-expressed cytokines and chemokines (Table S6), members of the IL-6 family, ligands and receptors, were up-regulated in both TME-stimulated CSCs and TME-stimulated Non-CSCs, each compared to “No stimulation”. (**B1**) Ligands of the IL-6 family that were up-regulated in TME-stimulated CSCs compared to “No stimulation”, and respective receptors for IL-6 ligands that were up-regulated in TME-stimulated Non-CSCs compared to “No stimulation”. (**B2**) Ligands of the IL-6 family that were up-regulated in TME-stimulated Non-CSCs and their respective receptors that were up-regulated in TME-stimulated CSCs. (**B3**) A scheme presenting possible ligand-receptor connections between IL-6 family members (based on the literature).

Moreover, when biological processes were determined by GO analyses of differentially-expressed genes (lists of enriched GO terms are detailed in Table S5) we found similarity in enriched processes in TME-stimulated CSCs and TME-stimulated Non-CSCs, when each group was compared to non-stimulated cells (Figure 3A). In both TME-stimulated CSCs and TME-stimulated Non-CSCs, up-regulated genes were highly associated with immunity and inflammatory-related processes, while down-regulated genes were enriched for cell cycle signatures. These analyses indicate that the effects of TME Stimulation on the cancer cell population, as a whole, lead to an increased immune/inflammatory phenotype and reduced cell proliferation.

GO term analyses of genes that were down-regulated in CSCs compared to Non-CSCs indicated that they included mainly gene signatures of processes related to development and differentiation, as well as processes related to adhesion (two lower panels in Figure 3A). Analyses of genes that were up-regulated in CSCs compared to Non-CSCs was not performed because of the very low number of genes that passed the FC and significance thresholds (8 genes passed the FC ≥ 2 and pFDR < 0.05 cutoffs, 20 genes passed the FC ≥ 1.5 and pFDR < 0.05 cutoffs and 21 genes passed the pFDR < 0.05 threshold, at any FC. These 21 genes are presented in Table S4). The findings concerning genes that were down-regulated in CSCs compared to Non-CSCs mark the TME Stimulation-derived CSCs as cells that are engaged in dedifferentiation, which is typical for cells shifting to the CSC state [14,55], and also having lesser adhesion properties, possibly reflecting reduced cell-to-cell contacts that characterize cells undergoing EMT [56].

If cross-talks indeed take place between TME-stimulated CSCs and TME-stimulated Non-CSCs, they most possibly can take form through interactions between ligands and receptors of soluble mediators. Indeed, cytokines and chemokines were particularly modified between TME-stimulated cells—CSCs and Non-CSCs, alike—and non-stimulated cells (Table S6). Particularly, the results indicated that members of the interleukin 6 (IL-6) network may mediate cross-talks between the TME-stimulated CSCs and TME-stimulated Non-CSCs, through the elevated expression of ligands and receptors of the family (Figure 3B1,B2). Figure 3B3 describes the possible interactions between the different members of the IL-6 pathway, ligands and receptors (based on the literature).

**Figure 4:**
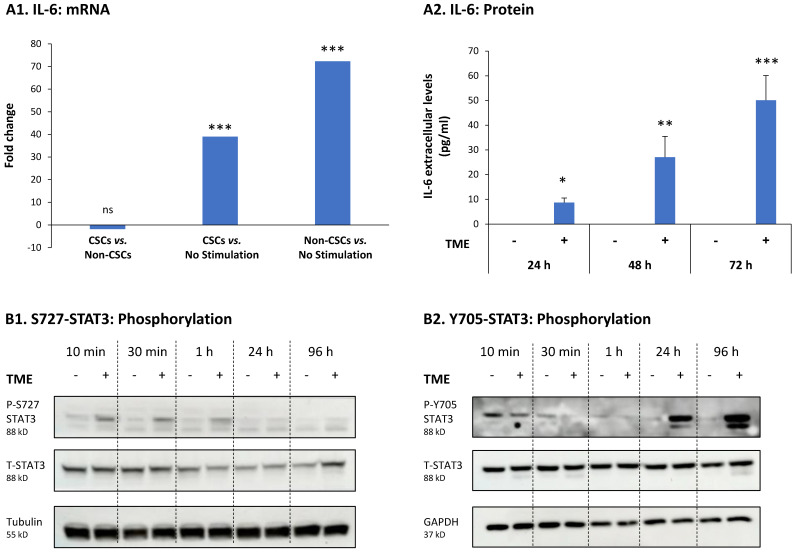
TME Stimulation leads to up-regulation of IL-6 expression, and to phosphorylation of STAT3 at the S727 and Y705 phosphorylation sites in HR+/HER2− breast cancer cells: MCF-7 cells were exposed to TME Stimulation (TME; Concentrations as described in Figure 1) or treated by a vehicle control (“No Stimulation”). (**A**) mRNA and protein levels of IL-6 after TME Stimulation. (**A1**) Fold change in IL-6 mRNA expression: left bar—non-significantly down-regulated IL-6 mRNA expression in TME-stimulated CSCs, when compared to TME-stimulated Non-CSCs; center bar—significantly up-regulated IL-6 mRNA expression in TME-stimulated CSCs, when compared to no stimulation; right bar—significantly up-regulated IL-6 mRNA expression in TME-stimulated Non-CSCs, when compared to no stimulation. IL-6 mRNA expression levels were determined by the RNAseq analyses described in Figure 2. (**A2**) Kinetics of IL-6 protein expression, along 24, 48 and 72 h of TME Stimulation, determined in CM of entire cell population by ELISA, at the linear range of absorbance (as explained in the Results section, this analysis could not have been performed on isolated CSC and Non-CSC subsets). (**B**) Phosphorylation kinetics of STAT3 at the S727 (**B1**) and Y705 (**B2**) sites, determined by WB analyses. In A2 and B, the results are from a representative experiment of *n* = 3, showing similar results. *** *p* < 0.001. ** *p* < 0.01. * *p* < 0.05. ns, not significant. The uncropped blots are shown in Figure S2.

Accordingly, we noted high mRNA expression levels of IL-6 in TME-stimulated CSCs and TME-stimulated Non-CSCs, when each of these two subsets was compared to non-stimulated cells (Figure 4A1; data derived from RNAseq analyses described in Figure 2). The production of IL-6 mRNA by the two TME-stimulated cell subsets was manifested by time-dependent elevations in IL-6 production at the protein level in the entire cell culture, being clearly noticeable at 48-72 h after the beginning of TME Stimulation (Figure 4A2; this analysis could not be performed on isolated CSCs and Non-CSCs because it would have required cell growth in culture; under these conditions, CSCs drift back to generate the entire cell population [16]).

Overall, the results obtained by the RNAseq analyses suggest that following TME Stimulation, CSCs and Non-CSCs are driven towards the expression of immunity/inflammation-related cell signatures and reduced cell proliferation; in parallel, TME Stimulation-driven CSCs exhibit characteristics of dedifferentiation and EMT-related signatures, a phenotype that may be connected to their stemness. Moreover, a network established between different IL-6 family members, ligands and receptors, may be taking place between the CSCs and Non-CSCs obtained following the stimulation of HR+/HER2− cells by the combined stimulus of estrogen + TNFα + EGF, representing three different arms of the TME of HR+/HER2− tumors.

### 3.3. STAT3 Regulates the Pro-Metastatic Activities of TME-Stimulated HR+/HER2− Breast Cancer Cells

The above-mentioned findings, suggesting involvement of IL-6 in TME Stimulation-driven effects in HR+/HER2− breast cancer cells, have led us to determine whether STAT3, the canonical transcription factor that is activated by IL-6 [57,58], has regulatory roles in this system. Thus, we next determined the kinetics of STAT3 activation, analyzing the phosphorylation of Y705-STAT3 which is considered a prime activation site of STAT3 [59,60,61], as well as of S727-STAT3 which is also involved in regulation of STAT3 activities [60].

The findings of Figure 4B1 Indicate that the S727-STAT3 site was phosphorylated already at 10 min after tumor cell stimulation, and its phosphorylation decayed at the late points of 24 and 96 h (Figure 4B1 and Figure S2); in parallel, the Y705-STAT3 site was phosphorylated at relatively slow kinetics, noted from 24 h post TME Stimulation and increasing at 96 h of TME Stimulation (Figure 4B2 and Figure S2). The relatively late activation of the Y705-STAT3 site agrees well with the kinetics of IL-6 protein production following TME Stimulation, suggesting that the Y705-STAT3 site was activated by IL-6 that has been starting to accumulate extracellularly after stimulation by TME factors.

**Figure 5:**
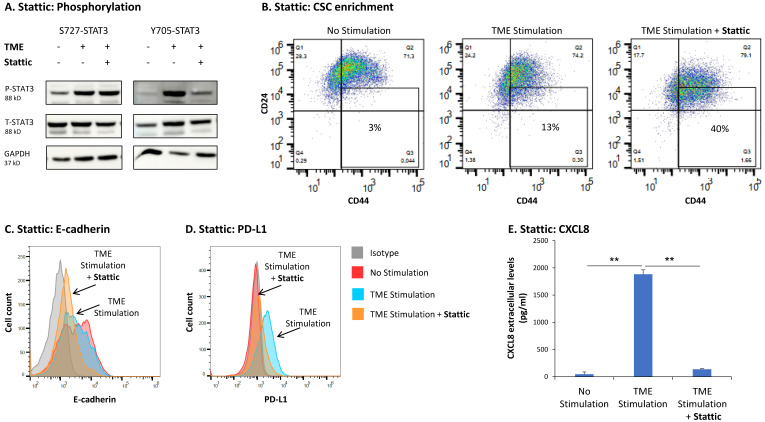
Upon TME Stimulation, the STAT3 inhibitor stattic leads to up-regulation of intrinsic pro-metastatic properties and to down-regulation of TME-related pro-metastatic traits of HR+/HER2− breast cancer cells: MCF-7 cells were exposed to TME Stimulation (TME; concentrations as described in Figure 1) or treated by a vehicle control (“No Stimulation”). Two h prior to stimulation and also during stimulation, the cells were incubated with STAT3 inhibitor stattic (5 μM, conventionally used concentration) or its vehicle. (**A**) The effect of stattic on S727-STAT3 (after 15 min of TME Stimulation, based on the findings of Figure 4B1) and Y705-STAT3 phosphorylation (after 96 h of TME Stimulation, based on the findings of Figure 4B2), were determined by WB analyses. (**B**–**E**) Functional effects of stattic, determined after 96 h of TME Stimulation, as described in Figure 1. In all panels, the results are from a representative experiment of *n* ≥ 3, showing similar results. ** *p* < 0.01. Results obtained from similar studies of CSC enrichment in T47D cells are demonstrated in Figure S1A. The uncropped blots are shown in Figure S2.

We then analyzed the impact of stattic, a commonly used STAT3 inhibitor (it binds to the STAT3 SH2 domain and inhibits its activation [62]), on TME Stimulation-induced pro-metastatic activities in HR+/HER2− breast cancer cells. The data of Figure 5A demonstrate that in MCF-7 cells, stattic potently inhibited the phosphorylation of the Y705 site of STAT3 but did not affect the phosphorylation of the S727 site (Figure 5A and Figure S2).

In terms of the effects of stattic on the functional properties of the cells upon TME Stimulation, we found that stattic potently increased the content of CSCs in TME-stimulated MCF-7 cells (Figure 5B); similar findings were detected in T47D cells (Figure S1-1A). In parallel, stattic considerably increased the TME Stimulation-induced EMT-like process in MCF-7 cells, identified by further reduced E-cadherin expression (Figure 5C). In contrast, stattic down-regulated the ability of TME Stimulation to potentiate the expression of PD-L1 and the release of CXCL8 by the cancer cells (Figure 5D,E, respectively). Thus, negative and positive roles were revealed for Y705-STAT3 activation in regulating TME-induced pro-metastatic effects in HR+/HER2− breast cancer cells.

**Figure 6:**
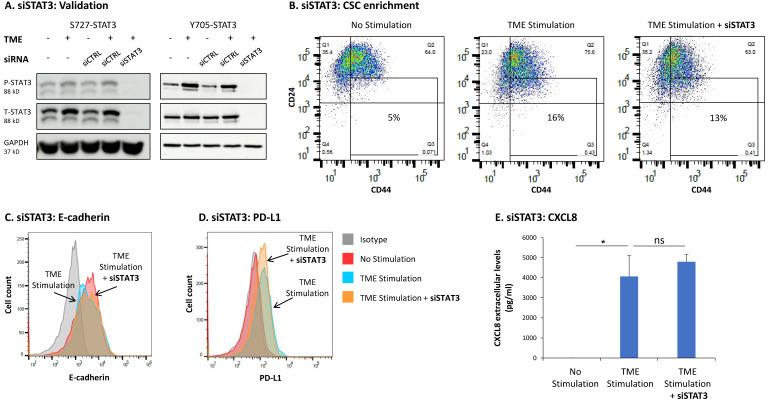
STAT3 knock-down does not affect the TME Stimulation-driven intrinsic and TME-related pro-metastatic traits of HR+/HER2− breast cancer cells: MCF-7 cells were transiently transfected with siRNA to STAT3 (siSTAT3) or control siRNA (siCTRL) and were exposed to TME Stimulation (TME; concentrations as described in Figure 1) or treated by vehicle control (“No Stimulation”). (**A**) The ability of siSTAT3 to down-regulate STAT3 expression and phosphorylation was determined after 96 h of TME Stimulation on Y705-STAT3 and S727-STAT3 phosphorylation, by WB analyses. (**B**–**E**) Functional effects of STAT3 KD, determined after 96 h of TME Stimulation, as described in Figure 1. In panels (**B**–**E**), the results are from a representative experiment of *n* ≥ 3, showing similar results. * *p* < 0.05. ns, not significant. Results obtained from similar studies of CSC enrichment in T47D cells are demonstrated in Figure S1B. The uncropped blots are shown in Figure S2.

Follow up studies that were performed by STAT3 knock-down (KD) with siRNA (validated in Figure 6A and Figure S2) pointed at a complex regulatory mechanism mediated by STAT3 following TME Stimulation. Unlike stattic (Figure 5), siSTAT3 did not affect any of the above-mentioned functions (Figure 6B–E). The proportion of CSCs, that was increased by stattic (Figure 5B in MCF-7 cells, and Figure S1-1A in T47D cells) was reduced significantly, although to a small extent, by siSTAT3 in MCF-7 cells (Figure 6B) and was not affected significantly in T47D cells (Figure S1-1B). In parallel, siSTAT3 only minimally affected the impact of TME Stimulation on the expression levels of E-cadherin and did not influence TME Stimulation-induced effects on PD-L1 and CXCL8 expression in MCF-7 cells (Figure 6C–E).

Together, our findings connect Y705-STAT3 activation to the negative regulation of intrinsic HR+/HER2− pro-metastatic properties (CSC, EMT-like) and positive control of functions that can affect the inflammatory/immune milieu at the tumor site (CXCL8, PD-L1 expression). Taking CSCs as a test case, our findings indicate that TME Stimulation increases the proportion of these cells to a certain level, which would have been further increased if Y705-STAT3 would not act as a negative regulator. However, when the entire STAT3 protein was lost (due to siSTAT3 use), with all of its regulatory domains and interactive connections with other proteins [59,60,61], it is possible that other transcription factor/s have kicked in and recapitulated STAT3 missing ability to down-regulate CSC enrichment upon TME Stimulation.

### 3.4. When the Entire STAT3 Protein Is Absent, Its Regulatory Effects Are Compensated by p65 Activation

To explore the possibility that the lack of the entire STAT3 protein was compensated by other transcription factor/s that simulated its activities, we investigated the effects of TME Stimulation on two additional transcription factors: STAT1 that under specific conditions is activated by IL-6 and can form heterodimers with STAT3 [61,63,64], and p65 which is the canonical transcription factor activated by TNFα [65,66], one of the factors included in TME Stimulation.

**Figure 7:**
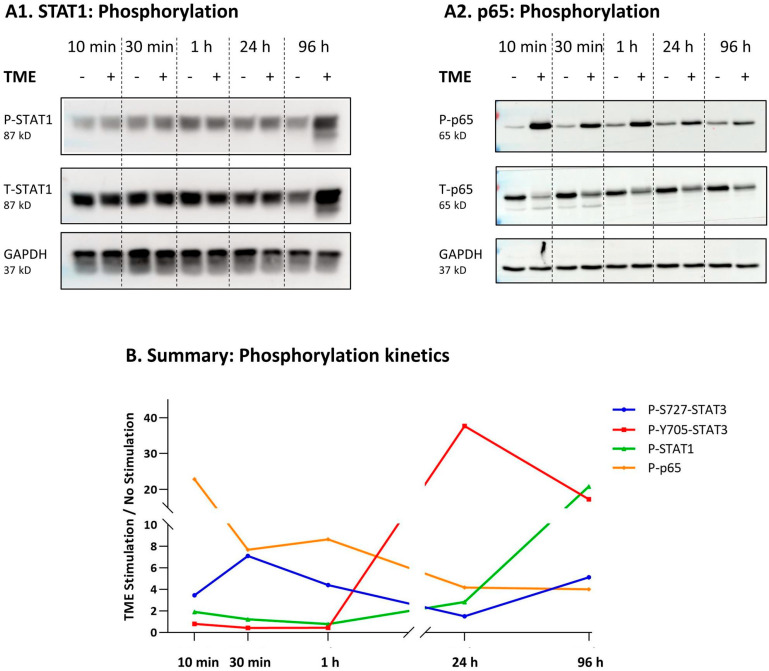
Upon TME Stimulation, STAT1 is activated in slow kinetics and p65 in rapid kinetics, in HR+/HER2− breast cancer cells: MCF-7 cells were stimulated by TME Stimulation (TME; concentrations as described in Figure 1) or treated by a vehicle control (“No Stimulation”). (**A**) Phosphorylation kinetics of STAT1 (**A1**) and p65 (**A2**), determined by WB kinetics analyses. The results are from a representative experiment of *n* = 3, showing similar results. (**B**) Summary of the kinetics of phosphorylation of S727-STAT3, Y705-STAT3 (representative experiments are shown in Figure 4), STAT1 and p65 (representative experiments are shown above in Figure 7A). The data in this part show the averages of *n* = 3 experiments for each phosphorylated protein, demonstrating fold change between TME-stimulated and non-stimulated cells. The uncropped blots are shown in Figure S2.

As shown in Figure 7A1,A2, TME Stimulation has activated both transcription factors but at different kinetics: the activation of STAT1 was detected 96 h after stimulation, whereas 10 min following exposure to TME factors, a strong activation of p65 was noted (Figure 7A and Figure S2). Of interest was the fact that the typical cytokines that induce STAT1 activation, namely interferon β (IFNβ; a representative of the two members of the Type I IFN family) and IFNγ (Type II IFN) [67] were not expressed by MCF-7 cells following TME Stimulation (Figure S1-2). In summary, TME Stimulation induced the activation of several transcription factors: S727-STAT3 and p65 were rapidly activated whereas slow kinetics of Y705-STAT3 and STAT1 activation were noted (Figure 4B and Figure 7A; summary of phosphorylation kinetics is demonstrated in Figure 7B).

**Figure 8:**
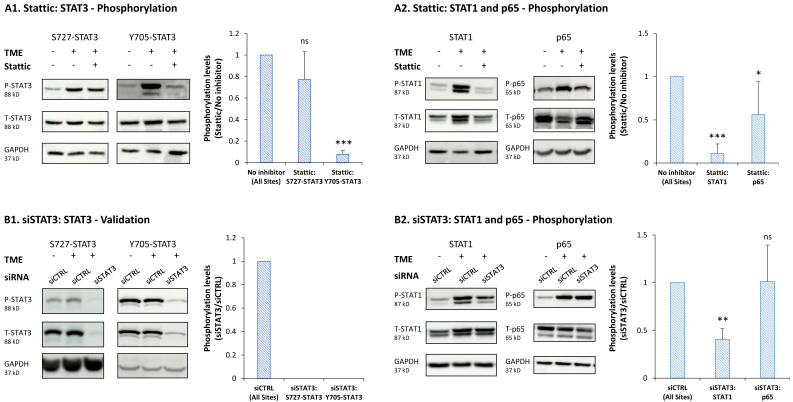
Upon TME Stimulation, stattic and siSTAT3 differently affect STAT1 and p65 activation in MCF-7 cells: the effects of the STAT3 inhibitor stattic and of STAT3 KD by siSTAT3 on STAT3, STAT1 and p65 activation were determined in MCF-7 cells that were exposed to TME Stimulation (TME; concentrations as described in Figure 1) or treated by a vehicle control. (**A**) The cells were incubated with stattic (5 μM) or its vehicle for two h prior to TME stimulation and also during stimulation (as described in Figure 5). (**A1**) Phosphorylation levels of S727-STAT3 and Y705-STAT3. The phosphorylation levels of S727-STAT3 were determined after 15 min of TME Stimulation, by WB analyses (based on the findings of Figure 4B1); Y705-STAT3 phosphorylation levels were determined after 96 h of TME Stimulation, by WB analyses (based on the findings of Figure 4B2). (**A2**) STAT1 and p65 phosphorylation levels. The phosphorylation levels of STAT1 were determined after 96 h of TME Stimulation, by WB analyses (based on the findings of Figure 7A1); the phosphorylation levels of p65 were determined after 15 min of TME Stimulation, by WB analyses (based on the findings of Figure 7A2). In all panels, the blots demonstrate the results of a representative experiment of *n* = 3, showing similar results. (**B**) The cells were transfected with siCTRL or siSTAT3 (as described in Figure 6). (**B1**) Phosphorylation levels of S727-STAT3 and Y705-STAT3. For both sites, phosphorylation levels were determined after 30 min of TME Stimulation, by WB analyses (and also after 96 h of TME Stimulation, as shown in Figure 6A). (**B2**) STAT1 and p65 phosphorylation levels. STAT1 phosphorylation levels were determined after 96 h of TME Stimulation, by WB analyses (based on the findings of Figure 7A1); p65 phosphorylation levels, determined after 15 min of TME Stimulation, by WB analyses (based on the findings of Figure 7A2). The STAT1 and p65 blots demonstrate the results of a representative experiment of *n* = 3, showing similar results. In all panels, the bar graphs demonstrate an average ± SD of *n* = 3 experiments for each phosphorylated protein, demonstrating fold change between stattic/siSTAT3 and control cells. *** *p* < 0.001. ** *p* < 0.01. * *p* < 0.05. ns, not significant. The uncropped blots are shown in Figure S2.

At this point, we have determined the effects of stattic and siSTAT3 on the activation of STAT1 and p65, each at the time of its activation peak. As demonstrated above in Figure 5A, stattic has considerably reduced the phosphorylation of Y705-STAT3 but did not have a significant effect on S727-STAT3 activation (for the sake of simplicity, results on S727-STAT3 and Y705-STAT3 phosphorylation are demonstrated again in Figure 8A1 (and Figure S2); a different experiment than in Figure 5A is presented in Figure 8A1). In parallel, stattic completely reduced the activation of STAT1 and partly of p65 (Figure 8A2 and Figure S2).

Similar studies that were performed with siSTAT3 (validated in Figure 8B1 and Figure S2) revealed two important findings: (1) siRNA to STAT3 induced, as with stattic (Figure 8A1), the down-regulation of STAT1 activation (Figure 8B2 and Figure S2), indicating that the effects of stattic on STAT1 activation were not due to an off-target effect of the inhibitor. Together with the kinetics data demonstrating that Y705-STAT3 is already activated at 24 h after TME Stimulation (Figure 4B2) and STAT1 is activated later (96 h), these findings suggest that Y705-STAT3 activation by TME Stimulation leads to the activation of STAT1. (2) Stattic and siSTAT3 affected the activation of STAT1 and p65 differently (Figure 8A2 vs. Figure 8B2). Stattic has led to total impairment of STAT1 activation, but siSTAT3 has induced only partial inhibition of STAT1 phosphorylation. In parallel, p65 phosphorylation was left only partly intact following stattic use, but remained fully active when siSTAT3 was used.

These findings indicate that when siSTAT3 was used and the expression of the entire STAT3 protein was down-regulated, it was accompanied by partial activity of STAT1 and full activation of p65; this pattern is different from the one obtained following stattic use, where loss of STAT1 activation and partial inhibition of p65 activation were noted. Thus, under the conditions of siSTAT3 use, STAT1 and/or p65 may compensate for the loss of STAT3 expression and recapitulate its activities. If this is indeed the case, when siSTAT3 is employed, the regulatory system is “ignorant” of the loss of STAT3 activities and all TME Stimulation-induced functions—elevation in CSCs, EMT, CXCL8 and PD-L1—remain as they were when STAT3 was intact.

**Figure 9:**
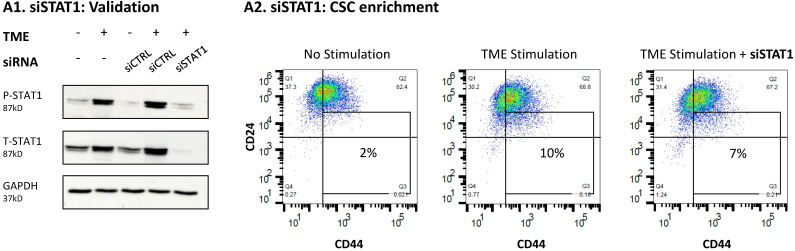
Upon TME Stimulation, STAT1 KD does not affect CSC enrichment in HR+/HER2− breast cancer cells: MCF-7 cells were transfected with siSTAT1 or siCTRL and have undergone TME Stimulation (TME; concentrations as described in Figure 1) or treatment by a vehicle control (“No Stimulation”). (**A1**) STAT1 phosphorylation levels. The ability of siSTAT1 to down-regulate STAT1 expression and phosphorylation was determined after 96 h of TME Stimulation. (**A2**) The contents of CSCs, determined following 96 h of TME Stimulation, as described in Figure 1. In Part (**A2**), the results are from a representative experiment of *n* = 3, showing similar results. The uncropped blots are shown in Figure S2.

**Figure 10:**
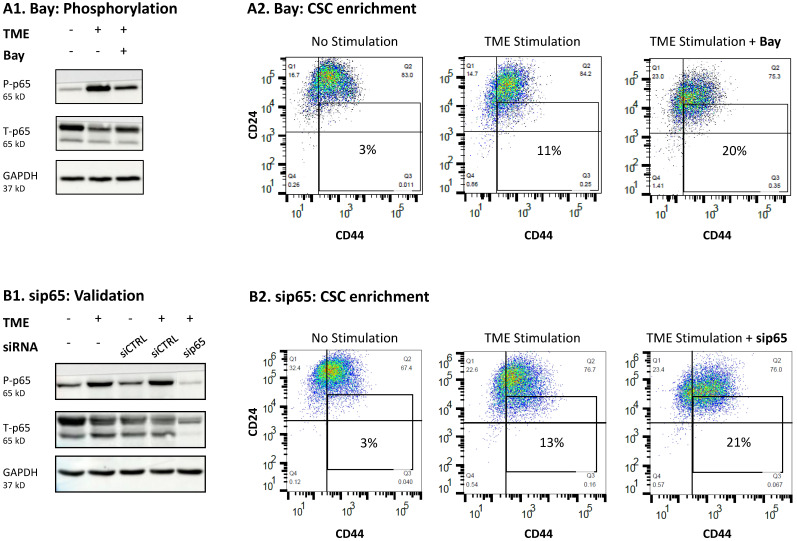
Upon TME Stimulation, p65 inhibition leads to CSC enrichment in HR+/HER2− breast cancer cells: in MCF-7 cells, the effects of the p65 inhibitor Bay 11-7082 (Bay) and of p65 KD by sip65 on CSC enrichment were determined upon TME Stimulation (TME; concentrations as described in Figure 1) or treatment by a vehicle control (“No Stimulation”). (**A**) The cells were incubated with Bay (5 μM, conventionally used concentration) or its vehicle for two h prior to TME stimulation and also during stimulation. (**A1**) p65 phosphorylation levels were determined after 15 min of TME Stimulation (based on the findings of Figure 7A2). (**A2**) The contents of CSCs, determined following 96 h of TME Stimulation, as described in Figure 1. In all panels, the results are from a representative experiment of *n* = 3, showing similar results. Results obtained from similar studies of T47D cells are demonstrated in Figure S1-3. (**B**) MCF-7 cells were transfected with sip65 or siCTRL. (**B1**) p65 phosphorylation levels were determined after 30 min of TME Stimulation (based on the findings of Figure 7A2). (**B2**) The contents of CSCs, determined following 96 h of TME Stimulation, as described in Figure 1. The results in Panel B2 are from a representative experiment of *n* = 3, showing similar results. The uncropped blots are shown in Figure S2.

To determine whether STAT1 and/or p65 can recapitulate the loss of the entire STAT3 protein (induced by siSTAT3), each of these transcription factors was inhibited; here, we expected that inhibition of either one of these transcription factors or both, would lead to the up-regulation of CSC levels in TME-stimulated cells, thus demonstrating that originally, they had negative regulatory roles in controlling CSC enrichment. First, our findings indicated that STAT1 did not serve as a regulator in this system, as revealed by the fact that CSC levels were not influenced by siSTAT1 (Figure 9 and Figure S2). In contrast, regulatory roles were revealed for p65 activation, as indicated by the use of the p65 inhibitor Bay 11-7082 (Bay; leading to 69–79% inhibition of p65 activation; a representative experiment is demonstrated in Figure 10A1 and Figure S2) and sip65 that has given rise to almost complete absence of p65 protein expression (Figure 10B1 and Figure S2). Both treatments resulted in elevations in CSCs levels upon TME Stimulation, thus demonstrating “protective” roles of p65 against elevated CSC levels (Figure 10A2,B2). The same analyses were performed with Bay in T47D cells, demonstrating similar results (Figure S1-3).

**Figure 11:**
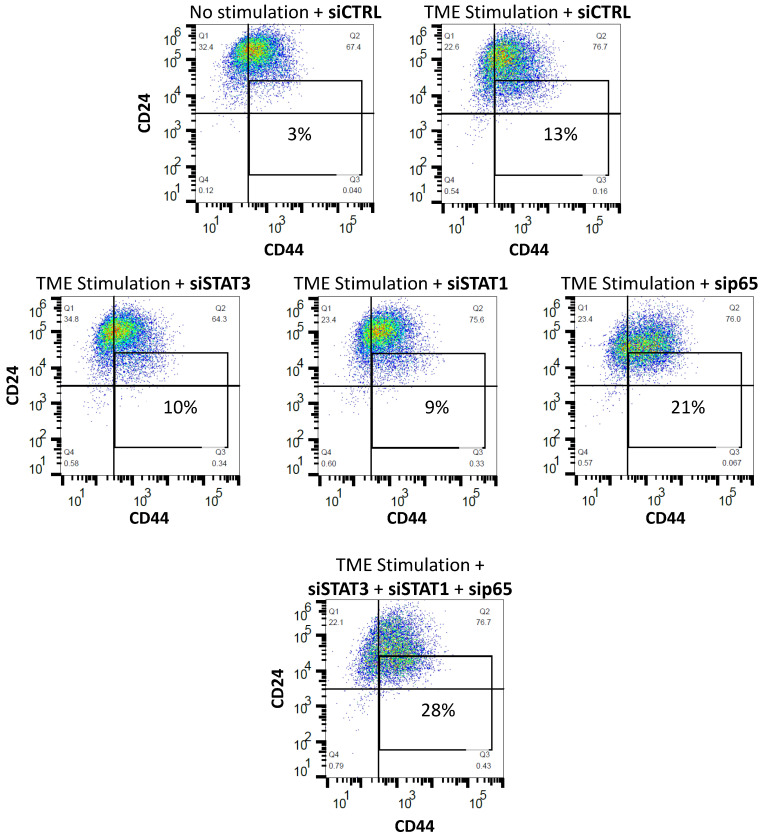
Upon TME Stimulation, knock-down of p65 and STAT3 together leads to further increased CSC enrichment in HR+/HER2− breast cancer cells: MCF-7 cells were transfected with siSTAT3, siSTAT1 and/or sip65 (or siCTRL), as described above. The cells were then exposed to TME Stimulation (concentrations as described in Figure 1) for 96 h or treated by vehicle control (“No stimulation”). The contents of CSCs were determined as described in Figure 1. The results are from a representative experiment of *n* = 3, showing similar results.

Further support to the activities of p65 in this network is provided in Figure 11, demonstrating the roles of each of the elements alone and together on CSC enrichment following TME Stimulation. Here, as in Figure 9 and Figure 10, siSTAT1 did not affect CSC levels and sip65 increased them. Moreover, combined use of siRNAs targeting STAT3 and p65 (and also STAT1—which did not have an effect when used alone, as noted above), has led to a further increase in CSC proportions upon TME Stimulation. The CSC elevation noted in this case was especially high, indicating that STAT3 and p65 have additive negative regulatory activities on CSC enrichment; thus, when the limiting functions of both these transcription factors were removed, further elevation in CSC levels were noted upon TME Stimulation, when compared to the down-regulation of each of them alone.

**Figure 12:**
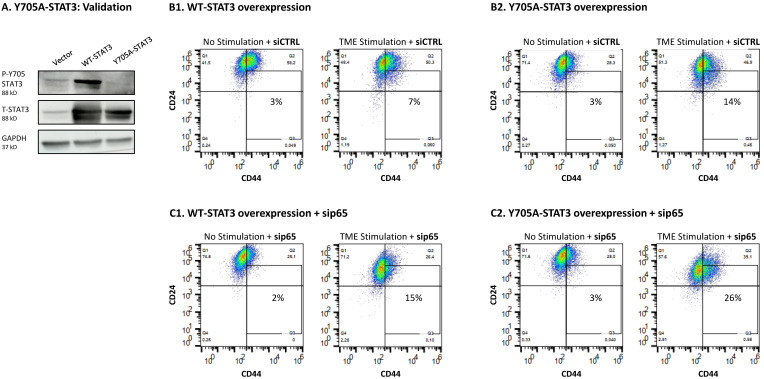
Upon TME Stimulation, mutated Y705A-STAT3 and p65 knock-down have additive functions, leading to further increased CSC enrichment in HR+/HER2− breast cancer cells: MCF-7 cells over-expressing WT-STAT3 or STAT3 mutated in Y705 (Y705A-STAT3) were generated. (**A**) Validation of reduced Y705-STAT3 phosphorylation in Y705A-STAT3 cells, determined after cell growth in culture. (**B**) Cells expressing WT-STAT3 (**B1**) or Y705A-STAT3 (**B2**) were transfected with siCTRL and were exposed to TME Stimulation (concentrations as described in Figure 1), or treated by vehicle control (“No Stimulation”). The contents of CSCs were determined following 96 h of TME Stimulation, as described in Figure 1. (**C**) Cells expressing WT-STAT3 (**C1**) or Y705A-STAT3 (**C2**) were transfected with sip65, and were analyzed similarly to the cells in Part B. The results in Parts B and C are from a representative experiment of *n* = 3, showing similar results. The uncropped blots are shown in Figure S2.

As a final approach to study the roles of STAT3 in the regulation of CSC levels, we followed up on the findings with stattic, which inhibited the phosphorylation only of Y705 in STAT3 (Figure 5A) and has increased CSC levels following TME Stimulation (Figure 5B). Here, we specifically targeted Y705 of STAT3 by generating cells over-expressing a STAT3 variant mutated at position Y705 (Y705A-STAT3) and compared them to cells over-expressing WT STAT3 (WT-STAT3) (Figure 12A and Figure S2). Following TME Stimulation, we noted an increase in CSC enrichment in Y705A-STAT3 cells compared to WT-STAT3 cells (Figure 12B1,B2). Of major interest was the fact that when p65 KD by sip65 has been introduced to Y705A-STAT3 expressing cells, an additive effect was obtained between the two measures, leading to further increased levels of CSCs in TME-stimulated cells (Figure 12C1,C2), when compared to each approach alone.

These findings indicate that of the two transcription factors, STAT1 and p65, p65 was the one having negative regulatory roles on CSC enrichment upon TME Stimulation. Its ability to act as down-regulator of CSC enrichment was revealed when it was solely inhibited by sip65 (Figure 10B) and more so when it was joined by inhibiting Y705-STAT3 through introducing the Y705A mutation. Under these latter conditions, p65 down-regulation by sip65 acted together with the mutated Y705A-STAT3 variant, leading to a further increase in CSC levels in TME-stimulated cells, when compared to the effects of each of them alone (Y705A-STAT3 or sip65) (Figure 11). This indicates a role for p65 in compensating for the loss of STAT3 expression, serving as a negative regulator of CSC enrichment upon TME Stimulation. Moreover, the findings of Figure 11 demonstrate additive functions of Y705-STAT3 and p65 in down-regulating CSC enrichment following the TME Stimulation of HR+/HER2− breast cancer cells.

### 3.5. The Negative Regulatory Roles of Y705-STAT3 + p65 on CSC Enrichment Have Clinical Relevance

In view of their general consideration as positive regulators of cancer progression, STAT3 and p65 are regarded as therapeutic targets in cancer [38,39,40,41,42,43]. Thus, the findings of our current study, demonstrating that these transcription factors negatively regulate CSC enrichment upon TME Stimulation of HR+/HER2− breast cancer cells, may have potential relevance in the clinic. In line with the current understanding that CSCs contribute to resistance of cancer cells to chemotherapy [68], we have previously shown that TME Stimulation-enriched CD44^+^/CD24^low/−^ cells had a survival advantage following doxorubicin treatment (that is employed in the clinic in the treatment of HR+/HER2− breast cancer patients [69]) of HR+/HER2− breast cancer cells [16], over non-stimulated cells.

**Figure 13:**
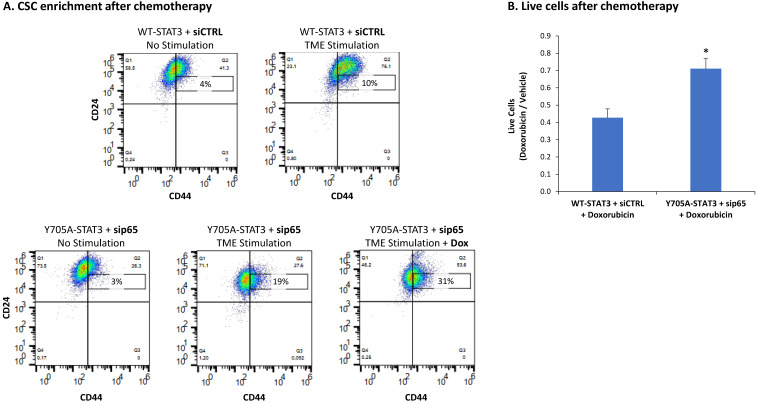
Upon TME Stimulation, the joint use of mutated Y705A-STAT3 and p65 knock-down leads to increased CSC resistance to doxorubicin in HR+/HER2− breast cancer cells: MCF-7 cells over-expressing WT-STAT3 or Y705A-STAT3 were transfected with sip65 or siCTRL. The cells were exposed to TME Stimulation (Concentrations as described in Figure 1), or treated by vehicle control (“No Stimulation”) for 96 h. During TME Stimulation or treatment by vehicle, the cells were also exposed to 0.5 µM doxorubicin (Dox), based on titration analyses. (**A**) The contents of CSCs were determined following 96 h of TME Stimulation, as described in Figure 1. CSC levels are presented in a narrower “window” than in previous figures, because doxorubicin induced elevation in autofluorescence of the cells, as reported previously by others [70]. The results are from a representative experiment of *n* = 3, showing similar results. (**B**) Average ± SD of *n* = 3 experiments demonstrating fold change between doxorubicin-treated and control cells treated by vehicle, based on cell counts using exclusion dye (trypan blue). * *p* < 0.05.

To follow up on these findings, we now examined whether doxorubicin treatment leads to increased CSC levels when the combined inhibition of STAT3 (Y705A-STAT3) and p65 (sip65) is employed. Following titration analyses in which doxorubicin cytotoxic effects were validated (See “Materials and methods”), we found that in cells lacking appropriate control of CSC generation due to Y705-STAT3 mutation + p65 KD, the presence of CSCs has been increased by doxorubicin treatment of TME-stimulated cells, compared to cells not treated by the drug (Figure 13A) (CSC percentages are presented in a narrower “window” than before, due to doxorubicin-induced elevation in autofluorescence of the cells, as previously described [70]). Moreover, cell counts at the end of TME Stimulation combined with doxorubicin has revealed that the entire cell population of Y705A-STAT3 cells with p65 KD showed higher resistance to chemotherapy than WT-STAT3 cells expressing normal p65 levels (Figure 13B).

**Figure 14:**
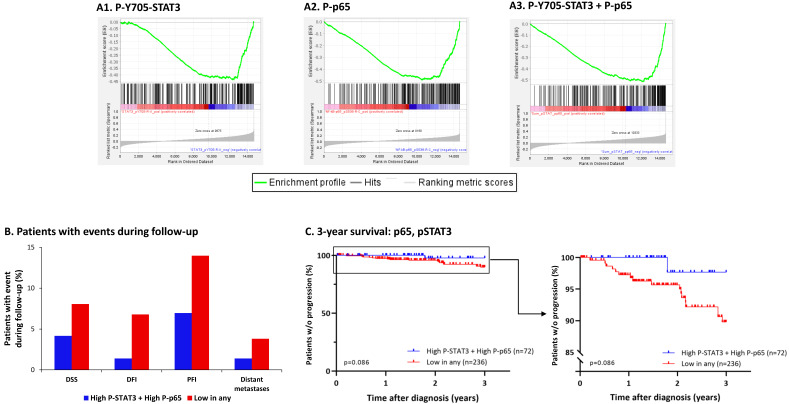
Clinical data of luminal A patients point at correlation of Y705-STAT3 activation + p65 activation with reduced CSC gene signatures and improved patient survival: mRNA, phosphoproteomics and clinical data of 308 samples of luminal A patients from the TCGA database were analyzed. (**A**) Gene set enrichment analyses (GSEA) for correlation between a CSC gene signature and (**A1**) P-Y705-STAT3 score; (**A2**) P-p65 score; or (**A3**) P-Y705-STAT3 + P-p65 score, are demonstrated. The CSC gene signature was created based on the significantly down-regulated genes in CSCs vs. Non-CSCs obtained in TME-stimulated MCF-7 cells (as described in Figure 2 and Table S1). (**B**) Incidence of patients (percentages) with event during the initial three years of follow-up, namely disease-free survival (DSS), disease-free interval (DFI), progression-free interval (PFI) and distant metastases. Patients were divided based on phosphoproteomics values: “High P-Y705-STAT3 + High P-p65”: patients at upper quartile for both phospho-sites (*n* = 72). “Low in any”: all other patients (*n* = 236). (**C**) Kaplan–Meier plot for survival analyses based on abundance of “High P-Y705-STAT3 + High P-p65” or “Low in any” patients, and PFI. The square outline shows enlargement of the upper part of the original plot.

To establish the clinical relevance of these in vitro results, further analyses were performed on breast cancer patient data from The Cancer Genomic Atlas (TCGA). TCGA data was extracted from data from primary breast tumors of patients categorized as luminal A according to their PAM50 subtype classification (mostly equivalent to the HR+/HER2− subtype). Patients were categorized based on (1) Y705-STAT3 phosphorylation score (termed herein “P-STAT3”); (2) p65 phosphorylation score (“P-p65”); or (3) Y705-STAT3 + p65 phosphorylation score, combined (“P-STAT3 + P-p65”). Expression data of the patients were then subjected to GSEA to determine enrichment for stemness gene signature (created based on the differential expression analyses of CSCs vs. Non-CSCs detailed in Figure 2 and Table S1). The results indicate that Y705-STAT3 and p65 phosphorylation in luminal A patients was inversely correlated with the gene signature of CSCs (Figure 14A). These analyses further support our hypothesis that Y705-STAT3 + p65 activation—each alone and together—inhibits CSC enrichment, now evidenced in luminal A patients.

Lastly, we determined the prognostic relevance of P-Y705-STAT3 and P-p65 by examining clinical events during the first three years of follow-up of the above-mentioned patients. Despite the relatively low number of patients in the TCGA database obeying the needs of this analysis, a tendency was evident towards a connection of high P-Y705-STAT3 + P-p65 levels in luminal A patients with lower incidence of disease progression, recurrence and metastasis, as well as with higher survival rates, when compared to all other luminal A patients together (Figure 14B,C).

Overall, these results emphasize the important role of Y705-STAT3 and p65 activation in keeping CSC levels in check, in the context of TME Stimulation. These data suggest a more complex role for STAT3 and p65 in CSC regulation than was envisioned before [71,72], suggesting that in parallel to their reported pro-tumorigenic properties, the activation of these two transcription factors may have protective roles under certain conditions, which may limit cancer progression.

## 4. Discussion

Tumor progression is regulated by the combined activities of many elements, including intrinsic tumor properties and cells/factors of the TME. Focusing on TME-derived factors, our previous findings have demonstrated the impact of a combined stimulus representing three key arms of the tumor milieu of HR+/HER2− tumors—including hormonal, inflammatory and growth stimulating factors—on the pro-malignancy phenotype of the cancer cells. This stimulatory setup was used as a model system to recapitulate the complex nature of the TME, which under this specific setup has induced in the cancer cells the enrichment in CSC proportions, which stood in the basis of increased metastatic potential of the tumor cells in vivo [16,34,35].

In the current study, we provided evidence to a large number of tumor-related phenotypes and functions that are induced by TME Stimulation in human HR+/HER2− breast cancer cells, MCF-7 and T47D. Overall, TME Stimulation has induced the strong enrichment of CSCs and has driven an EMT-like phenotype in the tumor cells (the latter only in MCF-7 cells). In parallel, much increased levels of CXCL8 and elevation in PD-L1 expression was induced in the cells by TME Stimulation (the latter, primarily in MCF-7 cells). Importantly, the levels of CSCs, EMT-like mesenchymal phenotypes, as well as the expression of CXCL8 and PD-L1, are generally low in luminal A patients and breast cancer cells [16,23,73,74,75,76,77]. Thus, our findings reveal a potential driving force—generated by a combination of TME factors—that pushes the cells into a more metastatic phenotype. These findings agree well with the observations on the transition of HR+/luminal breast cancer cells in patients to a more aggressive phenotype in the course of disease progression [5,6,7,8].

These observations have set the basis for the research of the molecular mechanisms involved in the pro-tumoral activities induced by TME Stimulation in HR+/HER2− breast cancer cells. The results of the current investigation have revealed novel findings on the functions of Y705-STAT3 in regulating TME Stimulation-driven effects on HR+/HER2− breast tumor cells, demonstrating that it has dual and opposing functions in controlling the pro-metastatic potential of the cells. First, through stattic experiments, we provided evidence for the roles of Y705-STAT3 as a negative regulator of tumor cell intrinsic properties that can potentiate metastasis, including enrichment of CSCs and EMT-like properties of the cells. This was evidenced by the fact that the reduced phosphorylation of STAT3 at the Y705 position by stattic has unleashed these pro-metastatic functions of the cancer cells, leading to the increased presence of CSCs and to an EMT-like phenotype in the cells. The negative regulatory roles of Y705-STAT3 on CSC generation was also evidenced by using the Y705A-STAT3 mutant, supporting the findings obtained with stattic.

Then, stattic experiments have demonstrated that Y705-STAT3 positively regulated the expression of mediators that can facilitate the ability of HR+/HER2− cells to use the TME for their own benefit: potentiating the inflammatory nature of the TME by increasing the levels of CXCL8, and down-regulating anti-tumor acquired immune activities by the elevated expression of PD-L1 by the tumor cells. These positive regulatory functions were evidenced by the fact that inhibition of Y705-STAT3 phosphorylation has potently reduced the TME Stimulation-driven increase in CXCL8 and PD-L1 expression.

In general, pro-tumorigenic effects were reported for STAT3 in malignancy, including in breast cancer, through the promotion of tumor cell proliferation, EMT, invasion and angiogenesis [38,39,40]. However, protective roles for the IL-6-STAT3 axis were also noted in cancer [78,79]. In the context of our current study, demonstrating the roles of Y705-STAT3 phosphorylation in protection against CSC enrichment and EMT, it is worth noting the connection of STAT3 to the maintenance of an epithelial phenotype in cancer cells. A recent study demonstrated that 94% of normal pancreatic tissues expressed STAT3 and in most cases Y705-STAT3 was moderately or strongly stained; in contrast, only 43% of pancreatic ductal adenocarcinoma samples expressed STAT3, which was generally weakly stained on the Y705 residue [80]. This study also demonstrated that consistent activation of STAT3 at the Y705 site conferred in prostate cancer cells a differentiated epithelial morphology, and that STAT3 loss induced a mesenchymal-like phenotype and higher tumor cell aggressiveness [80]. Moreover, a 2022 study of TNBC breast cancer patients demonstrated that high phosphorylation levels of Y705-STAT3 were associated with epithelial cell phenotype and luminal differentiation markers which are generally connected to lower levels of CSCs; in contrast, high phosphorylation levels of STAT3 at the S727 site were connected to a basal phenotype [81].

The observations of our current study also provided insights to the fact that in certain settings, the effects of STAT3 on malignancy may be regulated by the presence/absence of the entire protein, and not only at the Y705 activation level. Whereas Y705 phosphorylation has affected all pro-metastatic functions of TME-stimulated HR+/HER2− breast cancer cells, the complete absence of STAT3—with its S727 phosphorylation site and other regulatory domains [61]—had no influence on tumor-promoting activities, of any kind. Here, we found that with the lack of the entire STAT3 protein, p65 activation took its place, acting as a negative regulator of CSC enrichment (Figure 10 and Figure 11). Actually, p65 was found to have down-regulatory effects on CSC enrichment also independently of STAT3 absence (Figure 10). In this context, it was interesting to note that Y705-STAT3 and p65 had additive effects, as was revealed by further increased levels of CSCs when TME Stimulation was introduced in cells expressing a mutated Y705A-STAT3 and sip65 (Figure 11). Thus, not only STAT3 but also p65, which is considered a prime inducer of tumor inflammation with its most deleterious effects [43], can protect against CSC enrichment upon TME Stimulation, when the guardian roles of STAT3 are absent.

STAT3 is known to interact with many proteins, activators and repressors [61]. Accordingly, our findings reveal its interactive connections to p65 activities, in which p65 could compensate for the loss of the entire STAT3 protein and exert down-regulatory roles in terms of CSC enrichment upon TME Stimulation. In this respect, it is interesting to point out that cooperativity between Y705-STAT3 phosphorylation and p65 acetylation was required for generating anti-apoptotic and oncogenic events in cancer cells, as well as the induction of pro-inflammatory genes [82]. Physical interactions between STAT3 and p65 were also reported, leading to different transcription effects than interactions of STAT3 with p50 of the NF-κB pathway [83]. These findings suggest that the networks established in cancer cells between STAT3 and other molecules, in each specific setting, may be very important in regulating STAT3 activities.

Overall, we hereby reveal novel information on the functional consequences of Y705-STAT3 and p65 activation, and on the regulation of STAT3 activation at the expression level as well as through its post-translational modifications at the Y705 vs. the S727 sites, which have been described previously to have different effects of cell functions [81,84]. Eventually, the complex network established by STAT3 and p65 in regulating CSC enrichment upon TME Stimulation may have strong clinical implications, as we have noted by the studies on doxorubicin resistance and clinical data from luminal A patients. These studies have provided evidence for the roles of P-Y705-STAT3 + P-p65 in down-regulating the content of doxorubicin-resistant CSCs, and on the inverse correlation between P-Y705-STAT3 + P-p65 and CSC signatures in the patients. Moreover, high P-Y705-STAT3 + P-p65 levels were directly correlated with improved clinical parameters in the patients.

The TME Stimulation model that we have used in this study has combined the hormonal, inflammatory and growth-promoting arms of the TME; however other arrays of TME setups can be active in HR+/HER2− breast tumors, as well as in other malignancies. Thus, our findings emphasize the need to better identify the roles of STAT3 and its cross-talks with other transcription factors in each specific malignancy, in different TME settings and time points along tumor progression.

## 5. Conclusions

In the current study, we have recapitulated the intricate nature of the TME by stimulating HR+/HER2− breast cancer cells with a selected complex of factors, representing the hormonal, inflammatory and growth stimulatory arms of the TME. In this model system, we have identified key regulatory roles for STAT3 and of p65 activation in controlling TME Stimulation-driven pro-metastatic effects in HR+/HER2− breast tumor cells. In this respect, our research has provided insights to the mechanisms mediating the joint activities of TME factors, revealing a duality in Y705-STAT3 influence on pro-metastatic characteristics of the cancer cells, as well as the ability of p65 to act as an inhibitor of CSC enrichment and to back up the missing protective activities of STAT3 in terms of CSC generation.

These aspects of STAT3 functionality emphasize the need to determine in much detail and precision the particularities of STAT3 control in different cancer settings. The various regulators of STAT3 expression and the different inducers of its activation may lead to diverse functional effects, which may also be dictated by tumor intra- and inter-heterogeneity. Therefore, it is possible that under one set of conditions the tumor-promoting activities of STAT3 would dominate its cancer-inhibiting functions, whereas in others it would have the opposite effects.

Indeed, whereas most studies have identified STAT3 as positive regulator of cancer progression, other investigations pointed out that the IL-6/STAT3 axis can be a protective element in cancer [78,79]. More complex situations, such as the one revealed in our study, suggest that in a certain setting STAT3 could have opposing effects on the malignancy phenotype of the cells, and that under specific conditions its activities can be compensated by other cellular signaling mediators and transcription factors.

This complexity may lead to difficulties in using STAT3 as target for inhibition in cancer, adding to the inherent problems of high STAT3 sequence similarity to other members of STAT family, poor bioavailability and high toxicity of the currently proposed drugs. To date, in the clinic there are no selective inhibitors of STAT3 in use, either alone or in combination with other therapeutic measures, such as immunotherapy [40,85]. This situation calls for the consideration of other approaches that would modify STAT3 activities, for example targeting TME Stimulation-driven signals that control Y705-STAT3 activation. Specifically, in the context of the specific TME Stimulation setup that we have used in the present research, measures that inhibit estrogen receptors or TNFα are already in clinical use in malignancy or other diseases [86,87,88]; thus, one could envision their use under specific settings in cancer, which would require careful examination and study in years to come.

## Data Availability

Please see in “Materials and methods” section.

## Supplementary Materials

The following supporting information can be downloaded at: https://www.mdpi.com/article/10.3390/cancers15082255/s1, Figure S1-1: Upon TME Stimulation of T47D cells, the STAT3 inhibitor stattic and STAT3 knock-down have different effects on CSC enrichment; Figure S1-2: TME Stimulation does not lead to up-regulation of IFNβ or IFNγ expression in MCF-7 cells; Figure S1-3: Upon TME Stimulation of T47D cells, p65 inhibition leads to CSC enrichment in HR+/HER2− breast cancer cells. Figure S2: Uncropped blots relevant to all Western blot blots presented in the body of the manuscript and in Figures S1-1, S1-2 and S1-3; Table S1: Down-regulated genes in TME-stimulated CSCs compared to TME-stimulated Non-CSCs; Table S2: Comparison of TME-stimulated CSCs and non-stimulated cells: 60 top up-regulated genes and 60 top down-regulated genes; Table S3: Comparison of TME-stimulated Non-CSCs and non-stimulated cells: 60 top up-regulated genes and 60 top down-regulated genes; Table S4: Comparison of TME-stimulated CSCs and TME-stimulated Non-CSCs cells: All up-regulated genes and 60 most down-regulated genes; Table S5: List of GO terms enriched for differentially expressed genes between TME-stimulated CSCs, TME-stimulated Non-CSCs and non-stimulated cells; Table S6: Cytokines and chemokines that were differentially expressed in TME-stimulated CSCs, TME-stimulated Non-CSCs and non-stimulated cells.

## Abbreviations

CSCs, cancer stem cells; EGF, epithelial growth factor; EMT, epithelial-to-mesenchymal transition; HR+, hormone receptor-positive; PD-1, programmed cell death protein 1; PD-L1, programmed death-ligand 1; STAT, signal transducer and activator of transcription; TME, tumor microenvironment; TNFα, tumor necrosis factor α.
