# Supplementary material for: A Tumor Microenvironment-Driven Network Regulated by STAT3 and p65 Negatively Controls the Enrichment of Cancer Stem Cells in Human HR+/HER2− Breast Cancer
Source: Cancers (Basel). 2023 Apr 12;15(8):2255. doi: 10.3390/cancers15082255 (PMC10137120; doi:10.3390/cancers15082255)
Supplement: Supplementary file 1 [file cancers-15-02255-s001.zip › Ben-Baruch-Fig. S1-10.4.23.pdf]

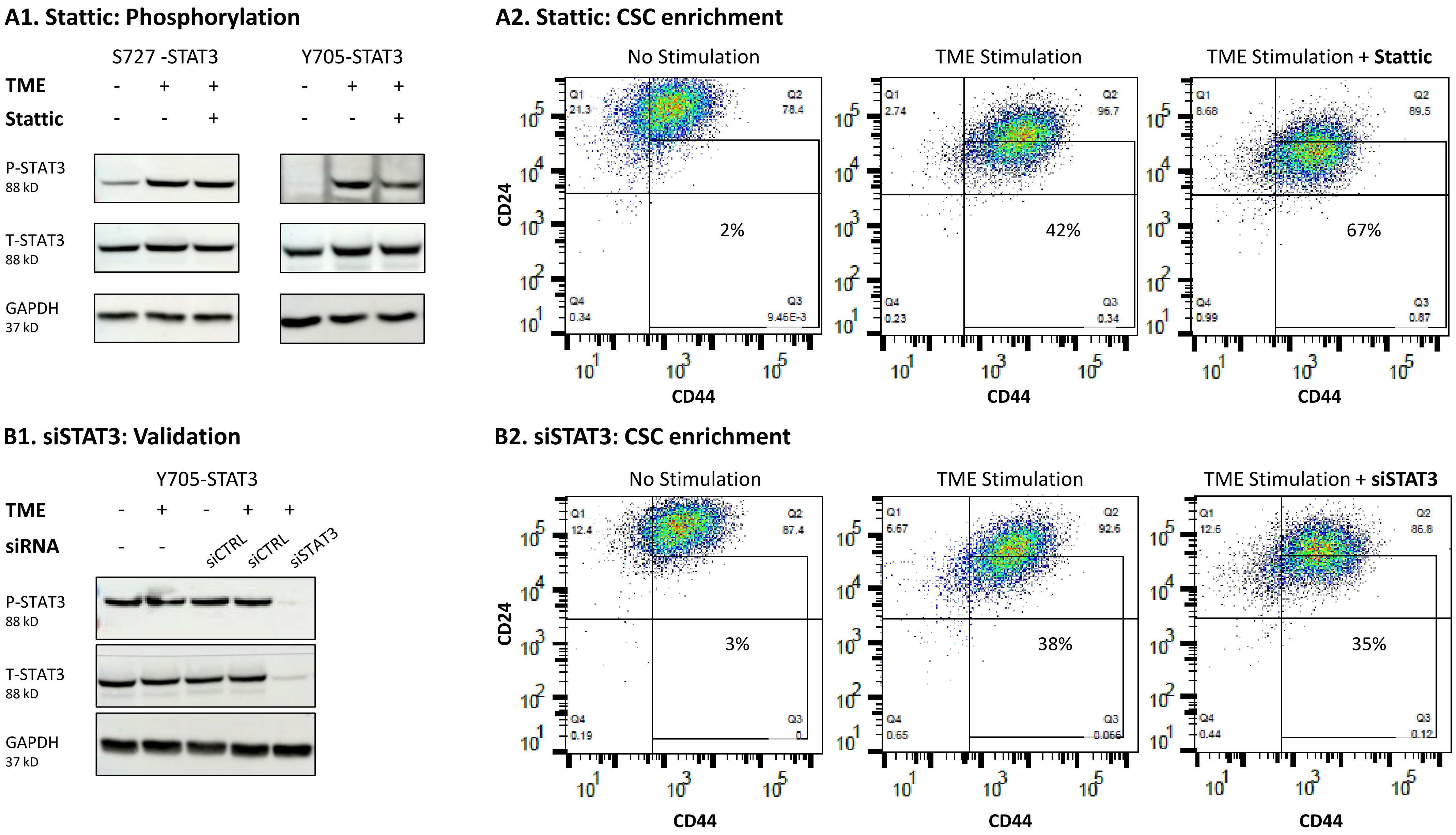

Figure S1-1

Upon TME Stimulation of T47D cells, the STAT3 inhibitor stattic and STAT3 knock-down have different effects on CSC enrichment

T47D cells were exposed to TME Stimulation (TME; Concentrations as described in Fig. 1), or treated by a vehicle control (“No Stimulation”). The effects of the STAT3 inhibitor stattic and of STAT3 knock-down on STAT3 phosphorylation and CSC enrichment were determined. **(A)** The effects of stattic. Two h prior to TME stimulation and also during stimulation, the cells were incubated with stattic (5µM, conventionally used concentration) or its vehicle. (A1) The effects of stattic on S727-STAT3 and Y705-STAT3 phosphorylation (after 15 min and 96 h of TME Stimulation, respectively, based on preliminary kinetics studies; Data not shown), were determined by WB analyses. (A2) The contents of CSCs, determined following 96 h of TME Stimulation, as described in Figure 1. In all panels, the results are from a representative experiment of n≥3, showing similar results. **(B)** The effects of siSTAT3. The cells were transiently transfected with siRNA to STAT3 (siSTAT3) or with control siRNA (siCTRL) (as described in “Materials and methods”) and were exposed to TME Stimulation (TME; Concentrations as described in Fig. 1), or treated by vehicle control (“No Stimulation”). (B1) The effects of siSTAT3 on Y705-STAT3 phosphorylation (after 30 min of TME Stimulation) and CSC contents (after 96 h of TME Stimulation) were determined as described in Part A, above. In Panel B2, the results are from a representative experiment of n≥3, showing similar results.

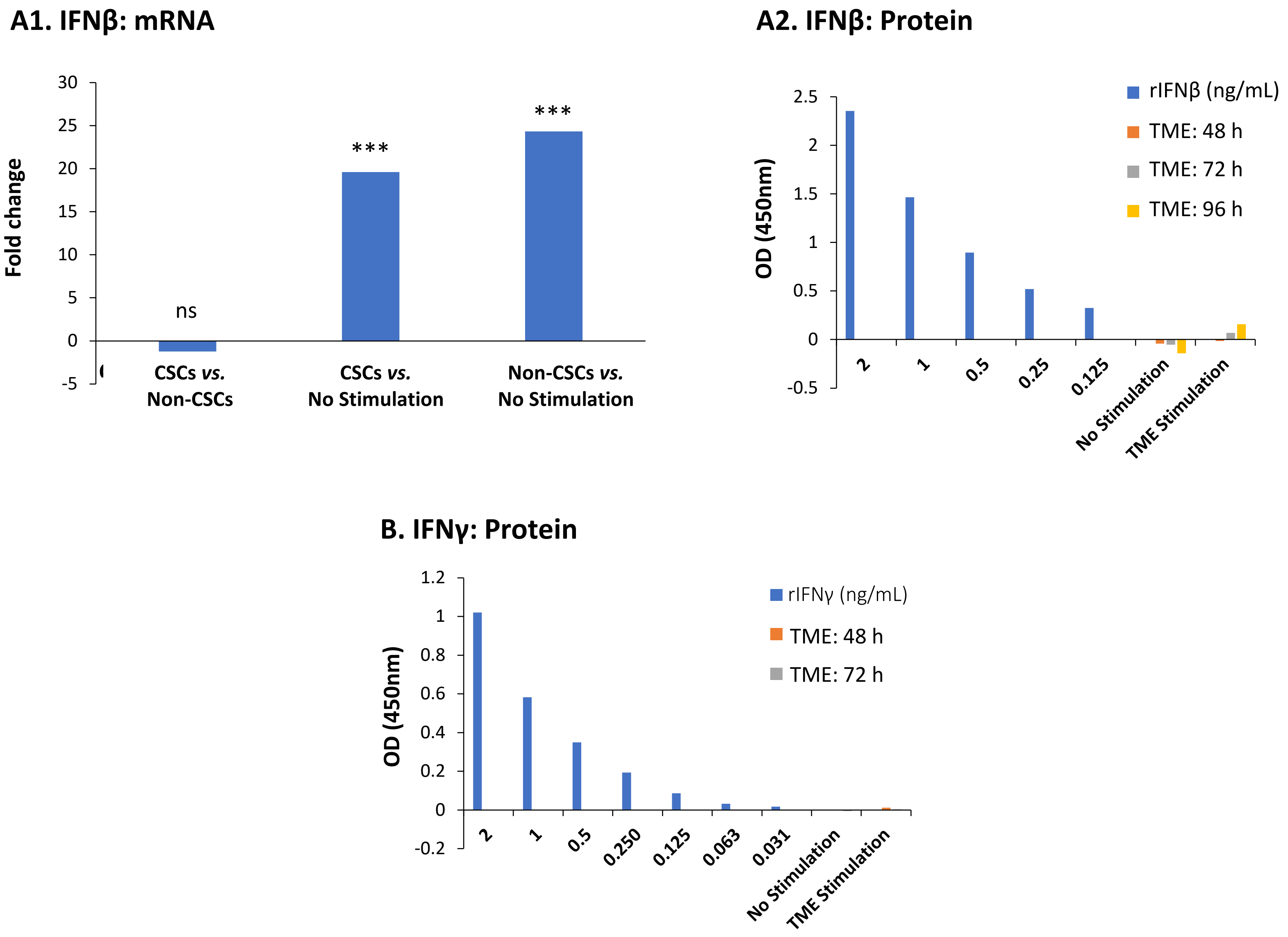

Figure S1-2

TME Stimulation does not lead to up-regulation of IFNβ or IFNγ expression in MCF-7 cells

MCF-7 cells were exposed to TME Stimulation (TME; Concentrations as described in Fig. 1) or treated by a vehicle control ("No Stimulation"). The levels of IFNβ as a representative of Type I IFNs **(A)** and of IFNγ as Type II IFN **(B)** were determined at the mRNA levels by the RNAseq analyses described in Figure 2, and/or at the protein levels in cell CM by ELISA. (A1) mRNA levels of IFNβ. (A2) IFNβ protein levels analyzed at different time points of TME Stimulation; the Figure also demonstrates the protein levels obtained with recombinant IFNβ (rIFNβ), used as positive control. (B) Protein levels of IFNγ, determined as described for Part A2; the Figure also demonstrates the protein levels obtained with recombinant IFNγ (rIFNγ), used as positive control. IFNγ mRNA expression was not detected at all by RNAseq in the TME-stimulated cells. ***$p<0.001$. ns, not significant.

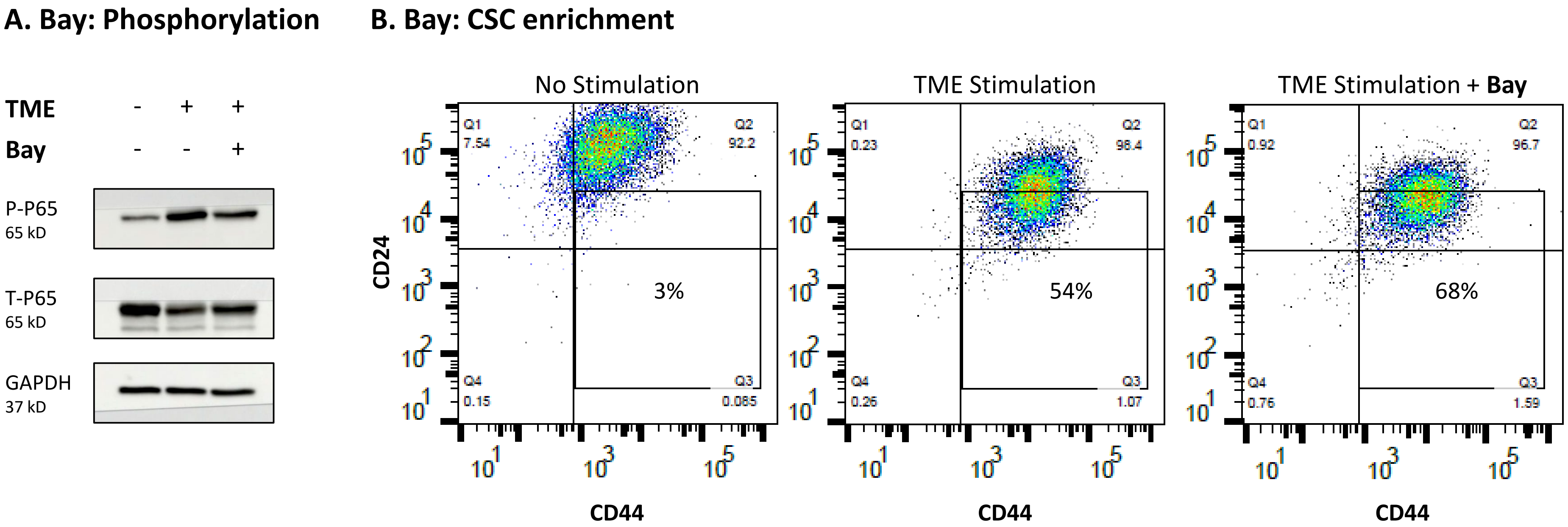

Figure S1-3

Upon TME Stimulation of T47D cells, p65 inhibition leads to CSC enrichment in HR+/HER2- breast cancer cells

In T47D cells, the effects of the p65 inhibitor Bay 11-7082 (Bay) on CSC enrichment was determined upon TME Stimulation (TME; Concentrations as described in Fig. 1), or treatment by a vehicle control ("No Stimulation"). The cells were incubated with Bay (5μM, conventionally used concentration) or its vehicle for two h prior to TME stimulation and also during stimulation. **(A)** The activation of p65 was determined by WB analyses (after 15 min of TME Stimulation, based on preliminary kinetics studies; Data not shown). **(B)** The contents of CSCs, determined following 96 h of TME Stimulation, as described in Figure 1. In all panels, the results are from a representative experiment of n≥3, showing similar results.
