# Supplementary material for: A Tumor Microenvironment-Driven Network Regulated by STAT3 and p65 Negatively Controls the Enrichment of Cancer Stem Cells in Human HR+/HER2− Breast Cancer
Source: Cancers (Basel). 2023 Apr 12;15(8):2255. doi: 10.3390/cancers15082255 (PMC10137120; doi:10.3390/cancers15082255)

WB raw data
# corresponding to Figure 4

S727-STAT3 - Phosphorylation

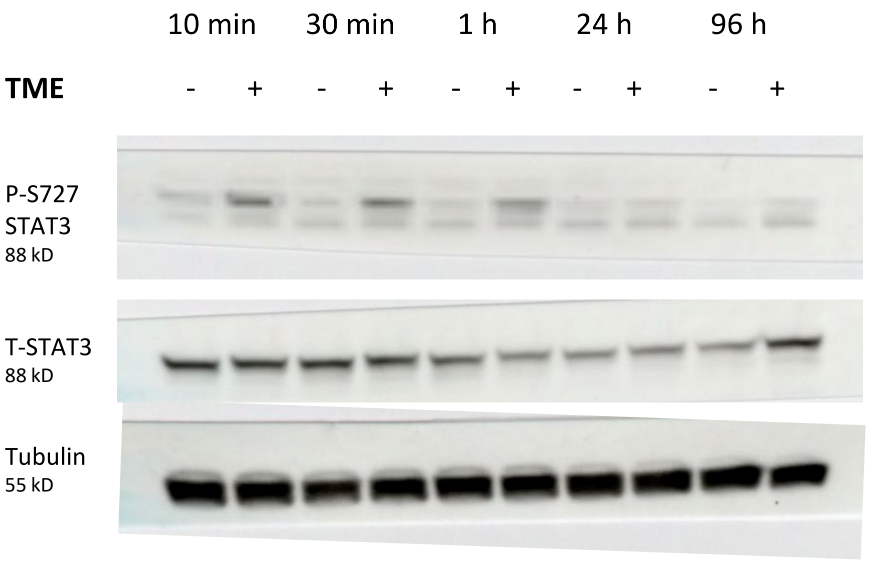

Y705-STAT3 - Phosphorylation

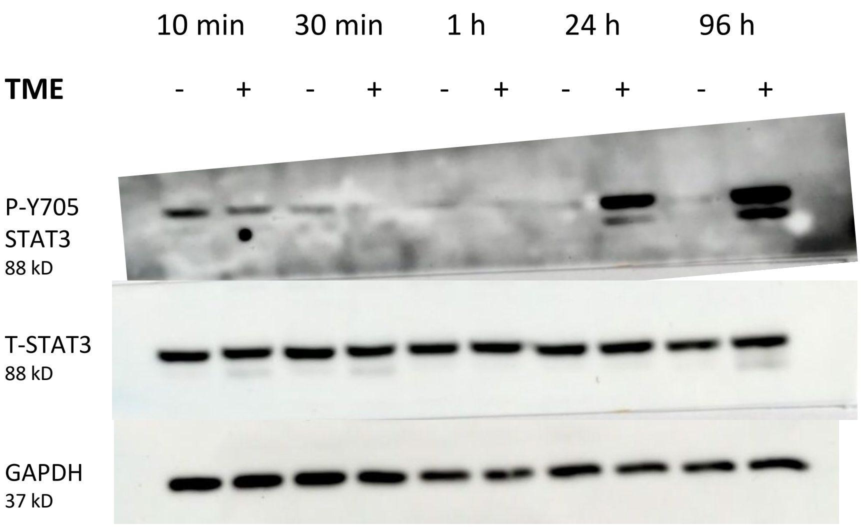

WB raw data
corresponding to Figure 5

S727-STAT3 - Stattic Validation

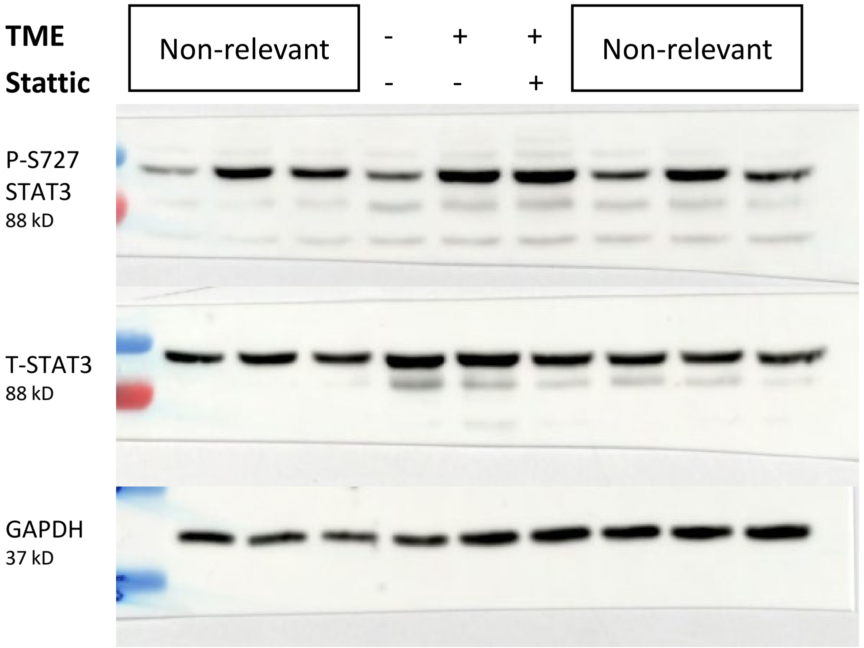

Y705-STAT3 - Stattic Validation

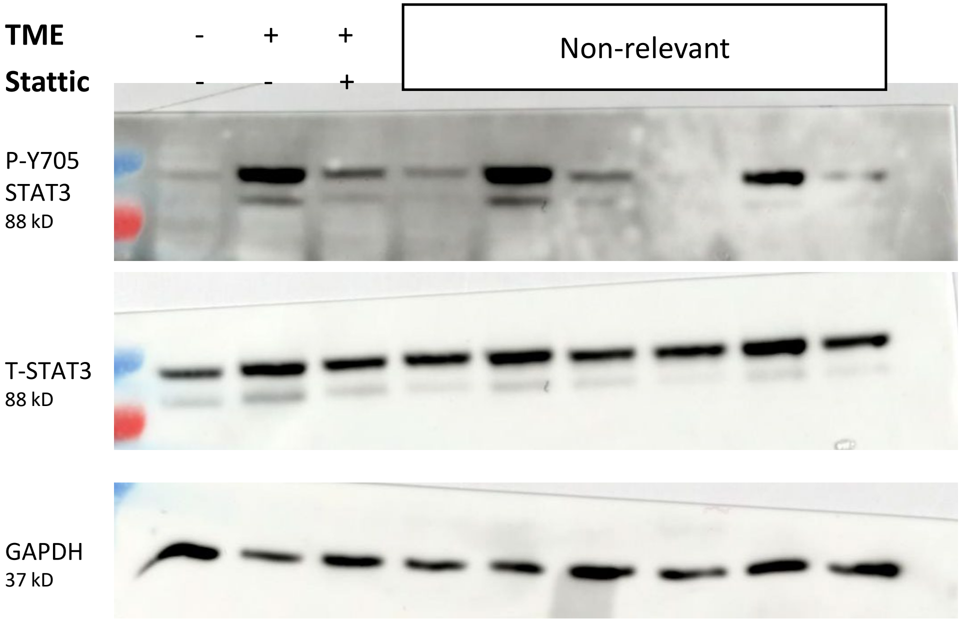

WB raw data
# corresponding to Figure 6

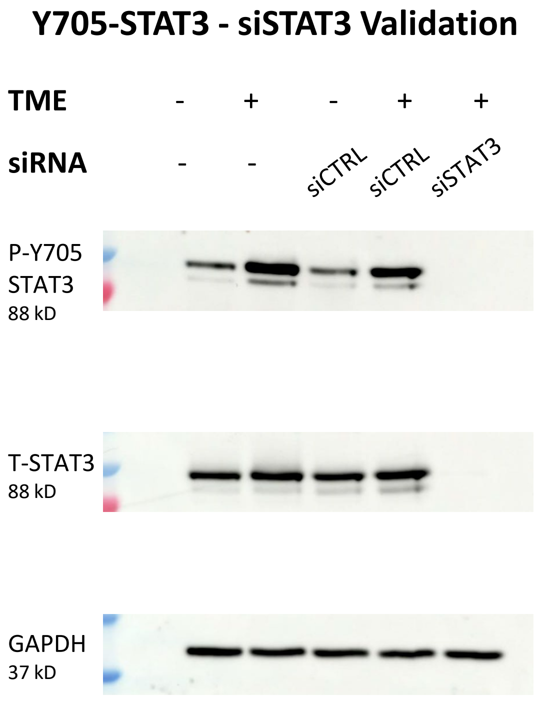

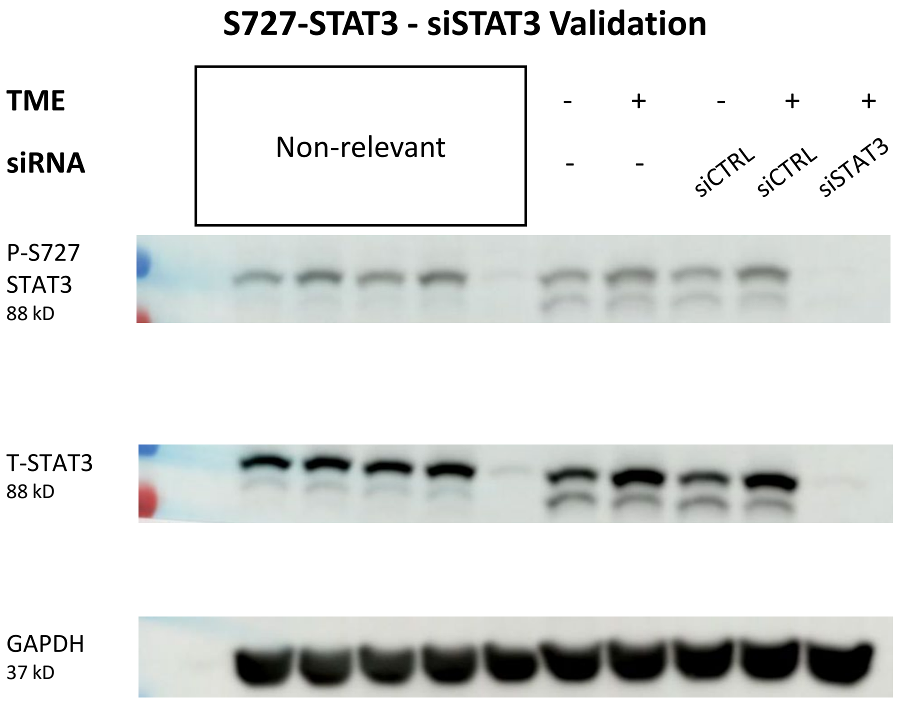

WB raw data
# corresponding to Figure 7

STAT1 - Phosphorylation

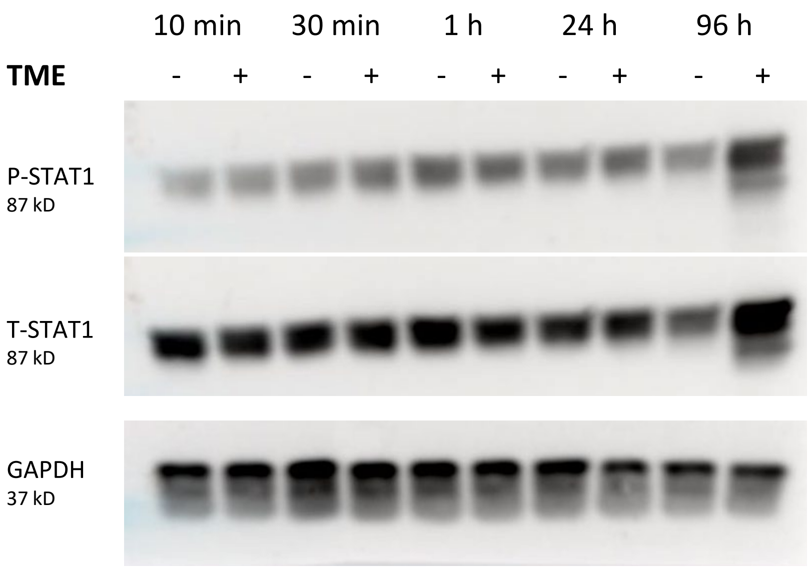

p65 - Phosphorylation

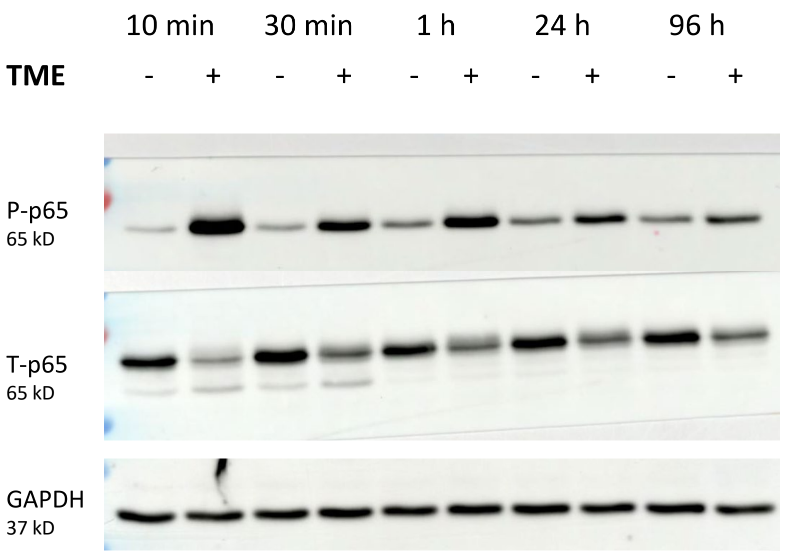

WB raw data
corresponding to Figure 8A

S727-STAT3 - Stattic

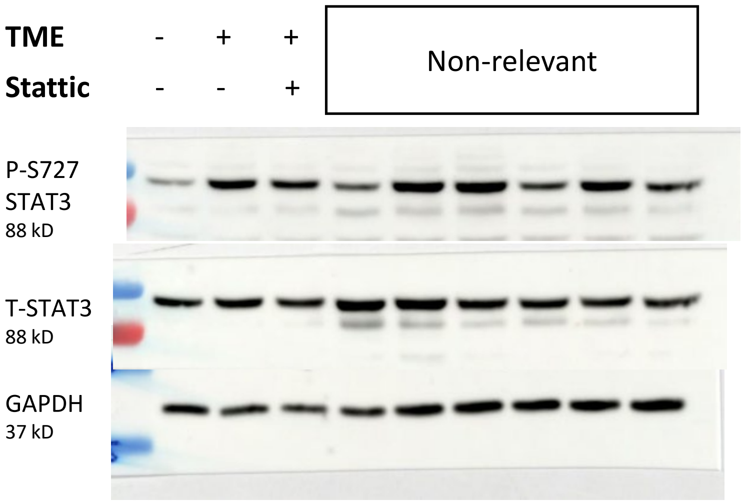

Y705-STAT3 - Stattic

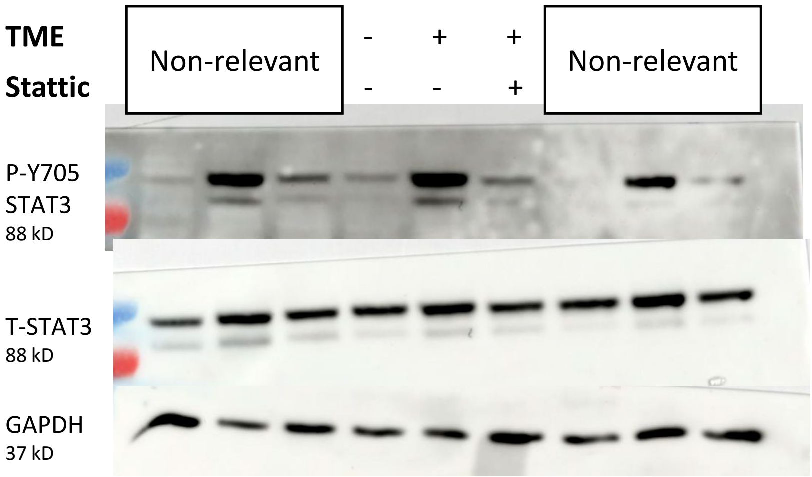

STAT1 - Stattic

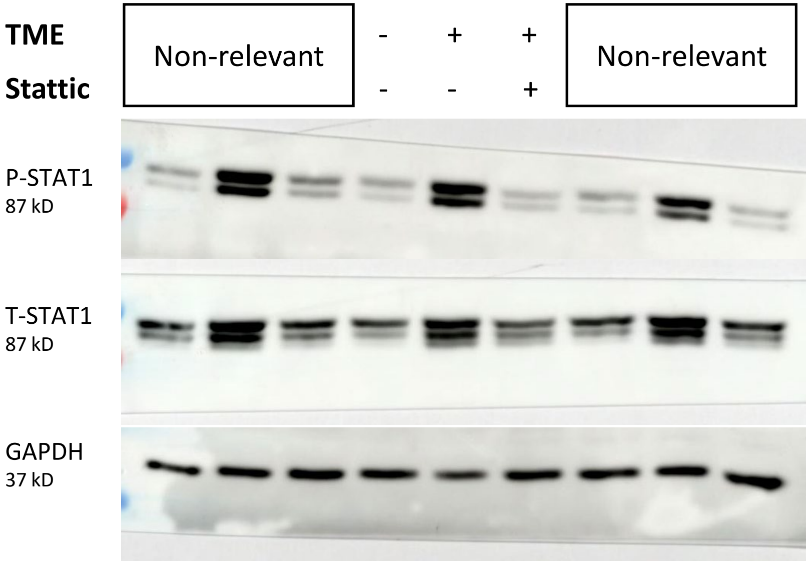

p65 - Stattic

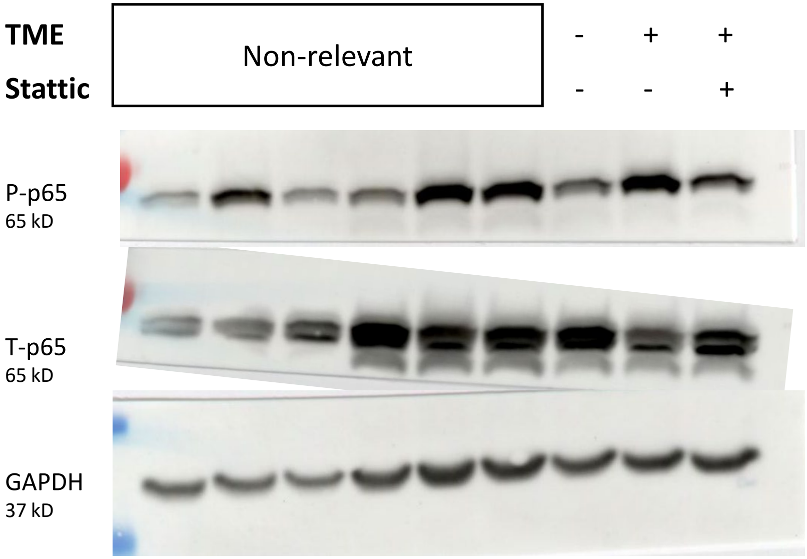

WB raw data corresponding to Figure 8B

S727-STAT3 - siSTAT3

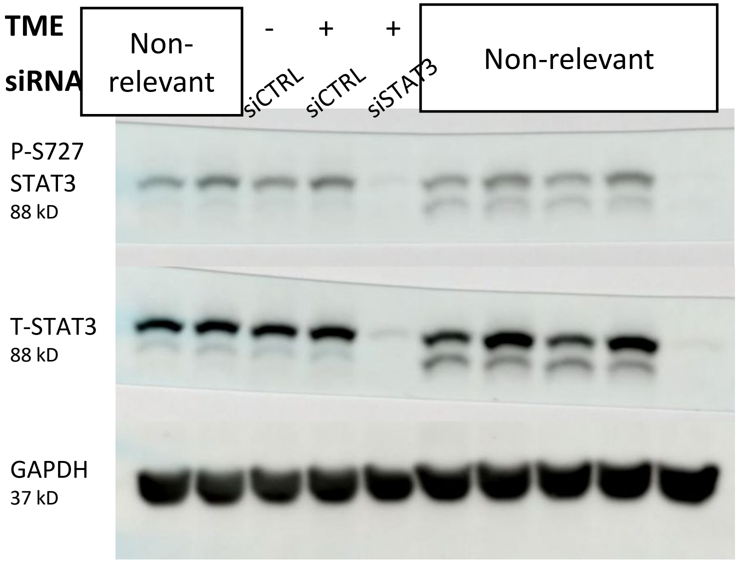

Y705-STAT3 - siSTAT3

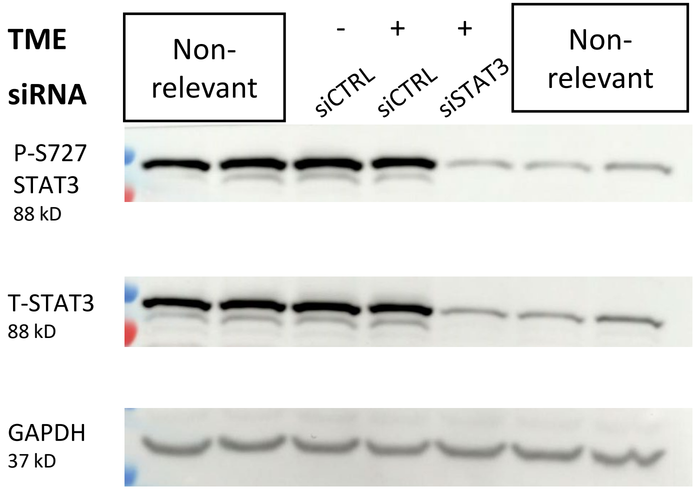

STAT1 - siSTAT3

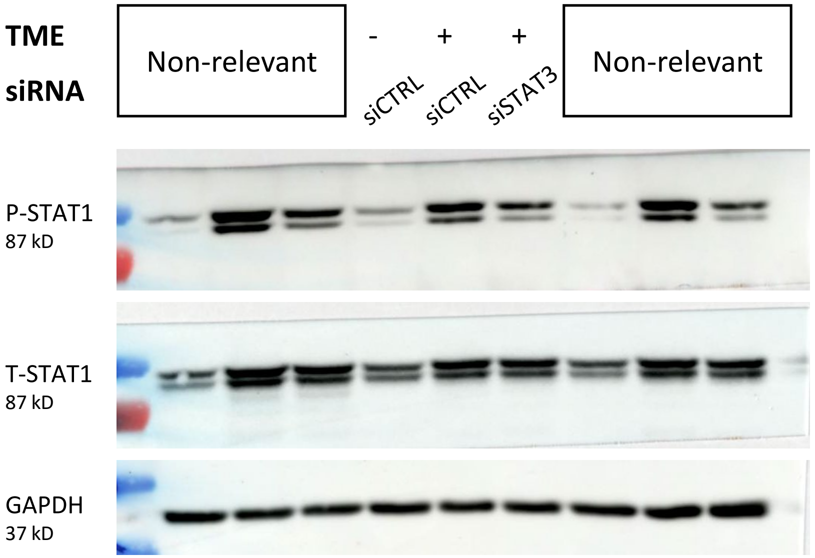

p65 - siSTAT3

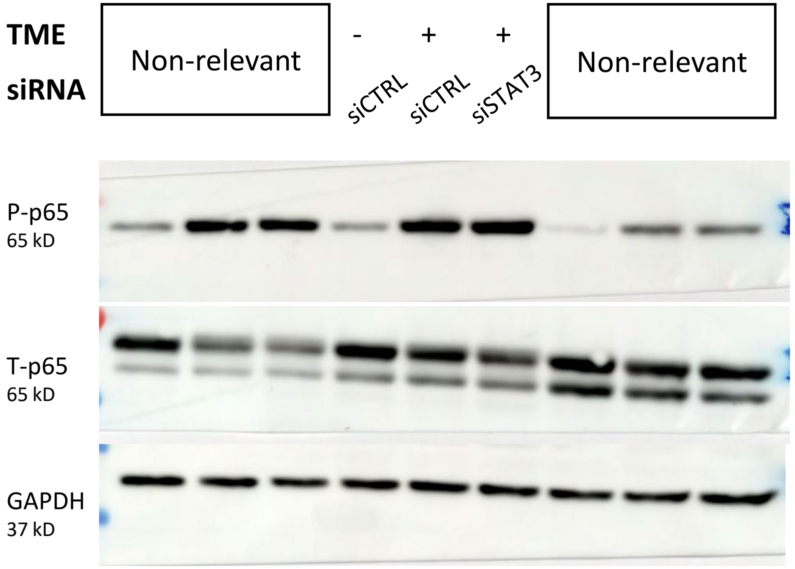

WB raw data corresponding to Figure 9

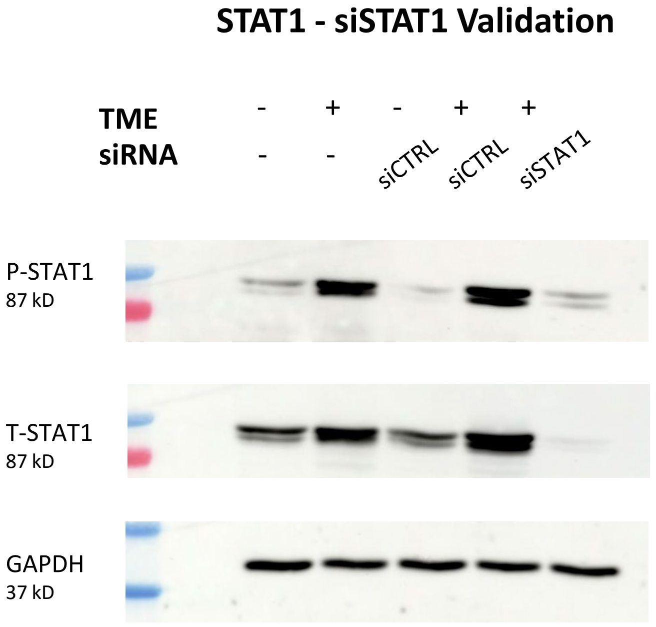

WB raw data
# corresponding to Figure 10

p65 - Bay Validation

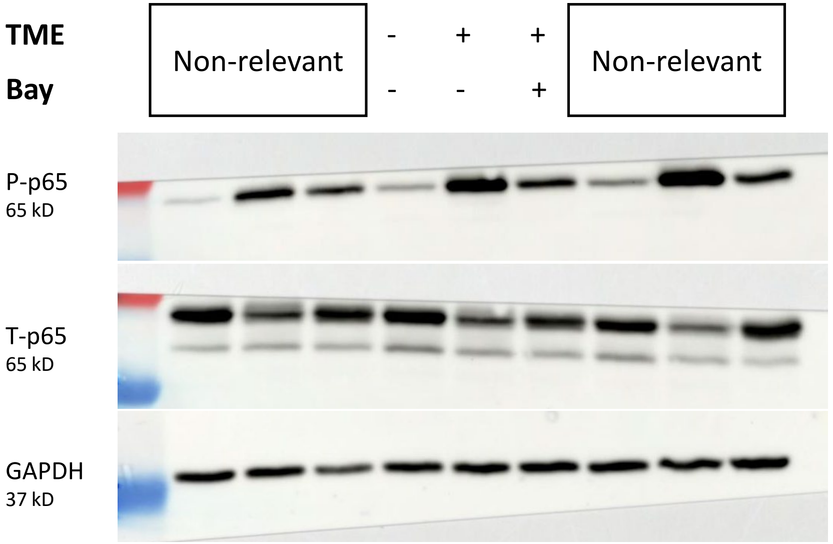

p65 - sip65 Validation

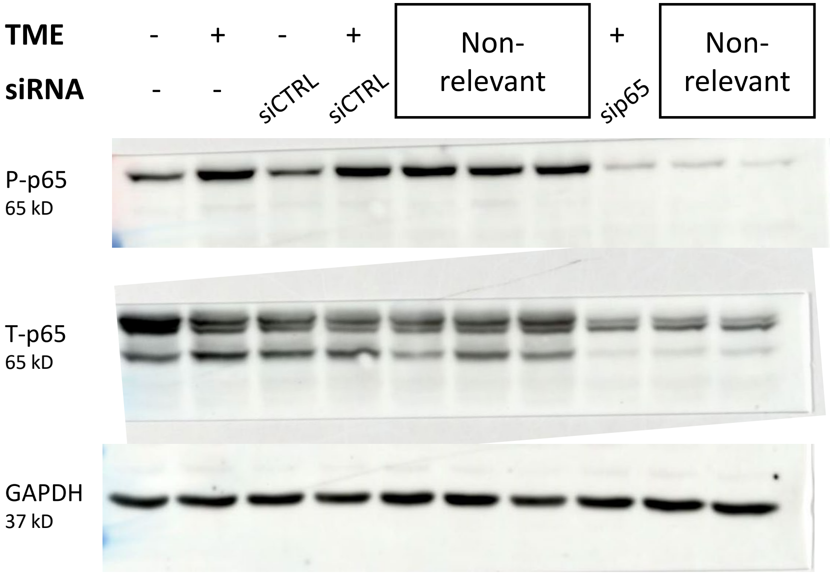

WB raw data corresponding to Figure 12

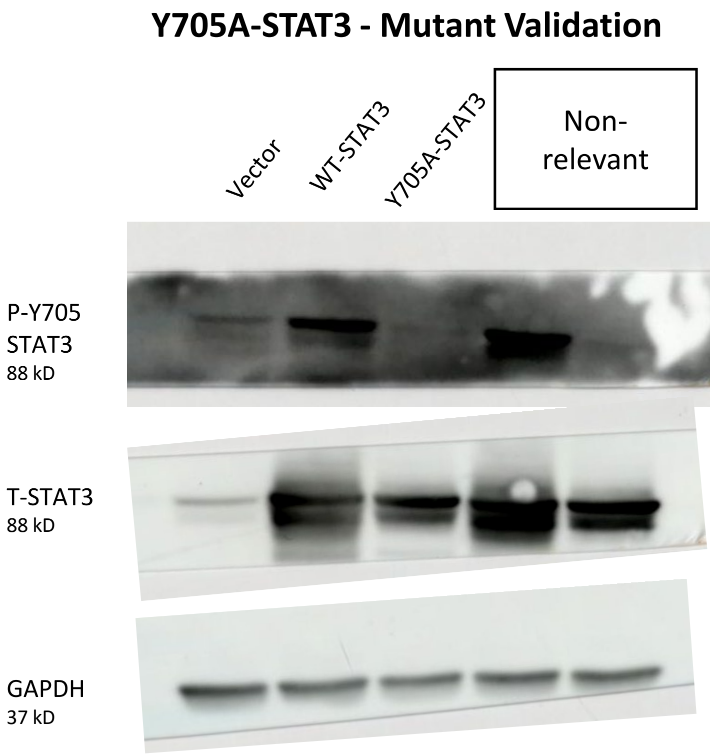

WB raw data
corresponding to Figure S1

S727-STAT3 - Stattic Validation

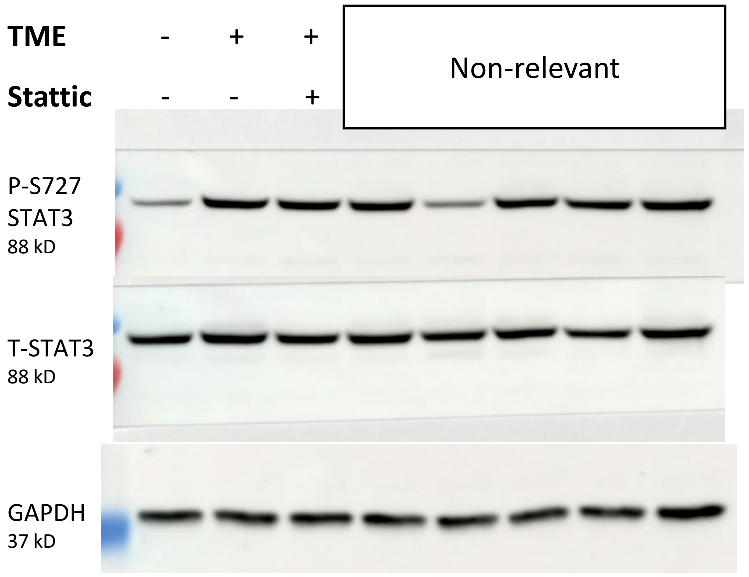

Y707-STAT3 - Stattic Validation

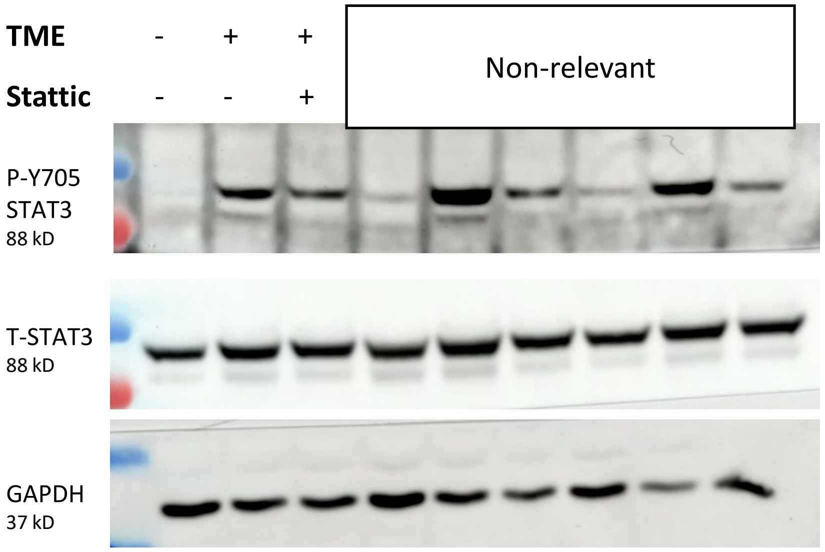

Y705-STAT3 - siSTAT3 Validation

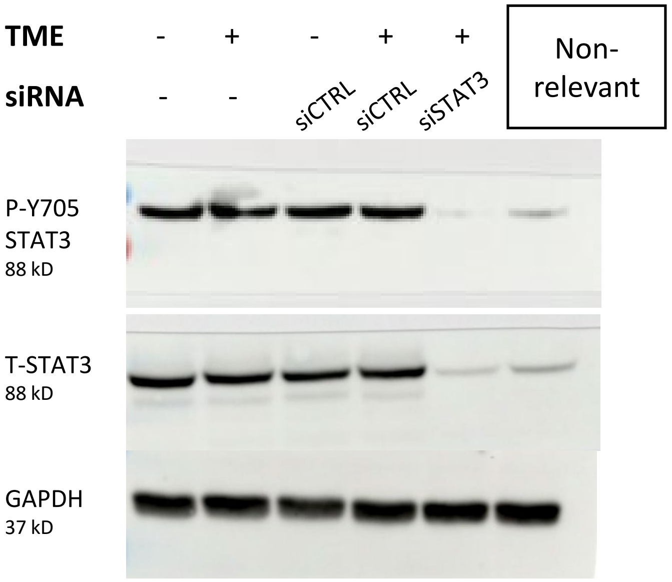

WB raw data
corresponding to Figure S3

p65 - Bay Validation

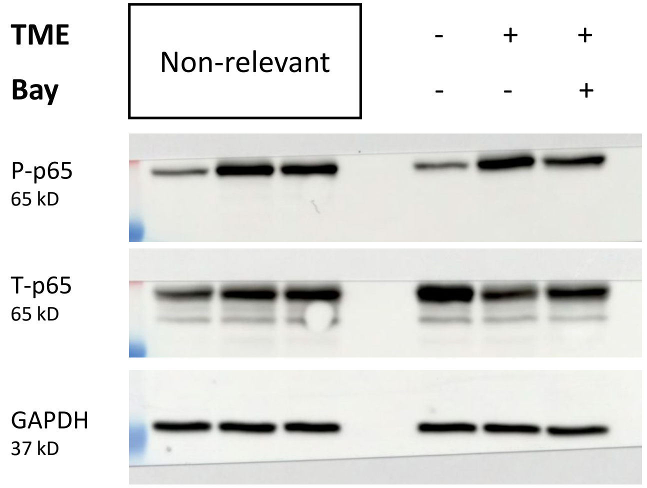

Supplement: Supplementary file 1 [file cancers-15-02255-s001.zip › Ben-Baruch-Fig. S2-10.4.23.pdf]
