# Supplementary material for: A Tumor Microenvironment-Driven Network Regulated by STAT3 and p65 Negatively Controls the Enrichment of Cancer Stem Cells in Human HR+/HER2− Breast Cancer
Source: Cancers (Basel). 2023 Apr 12;15(8):2255. doi: 10.3390/cancers15082255 (PMC10137120; doi:10.3390/cancers15082255)
Supplement: Supplementary file 1 [file cancers-15-02255-s001.zip › Ben-Baruch-Supplemenatry Tables-4.4.23.pdf]

A. Up-regulated genes

Gene	FDR step up	Fold change
BIRC3	2.E-53	78.12
LINC02015	3.E-41	63.41
SAMD9L	6.E-25	61.72
TCIM	3.E-23	56.02
S100A9	1.E-41	55.66
TLR6	1.E-14	39.23
IL6	6.E-13	38.99
GBP1	8.E-49	34.36
TRIM22	1.E-36	28.63
C1S	2.E-25	24.88
KLHDC7B	2.E-68	24.22
IRAK2	2.E-41	21.91
CTSS	1.E-14	21.48
C3	8.E-36	20.33
IFNB1	4.E-10	19.59
SLC28A3	6.E-22	19.42
CCL22	6.E-11	19.29
CCDC33	1.E-30	18.85
ITGAM	7.E-26	18.27
ARHGEF38	8.E-37	17.11
GBP4	5.E-19	16.71
STRA6	3.E-44	16.22
IFNL1	8.E-15	15.46
CXCL8	9.E-16	15.35
IFIT2	1.E-35	13.81
LCN2	2.E-15	13.11
C1R	5.E-21	12.76
AC006460.2	3.E-11	12.65
KRT17	4.E-16	12.31
PRAL	1.E-04	12.02

Gene symbol	FDR step up	Fold change
DAPP1	3.E-18	11.83
TRAF1	2.E-27	11.63
NPHS1	1.E-13	11.40
ERAP2	5.E-21	10.75
TNFAIP3	2.E-49	10.51
SOD2	6.E-52	10.37
AL391056.1	7.E-25	10.37
KRT8P18	2.E-08	9.82
C15orf48	5.E-15	9.70
AC013444.1	5.E-10	9.56
RHEBL1	1.E-15	9.51
BTN3A3	2.E-16	9.38
LAMC2	1.E-35	9.24
RSAD2	5.E-25	9.00
ICAM1	1.E-37	8.93
PTGES	4.E-19	8.92
IGFBP6	3.E-04	8.57
AC099521.2	2.E-08	8.40
MOB3B	2.E-09	8.36
AC004264.1	1.E-13	8.34
FGD6	8.E-38	8.05
MMP9	3.E-22	7.96
IFI44	2.E-22	7.91
CD74	1.E-14	7.74
SDR16C5	4.E-12	7.70
AC013444.2	8.E-18	7.59
C4orf54	6.E-12	7.58
OASL	3.E-19	7.41
LUCAT1	1.E-20	7.36
AC145098.2	4.E-38	7.34

B. Down-regulated genes

Gene	FDR step up	Fold change
RAMP3	2.E-73	153.06-
AC138811.2	9.E-43	110.27-
APOD	8.E-21	81.83-
IQGAP3	6.E-85	68.82-
HIST1H3B	6.E-77	66.42-
MYBL2	3.E-26	65.44-
SCUBE2	6.E-36	63.12-
UHRF1	4.E-58	56.83-
AC112777.1	1.E-32	55.87-
MKI67	2.E-106	55.53-
CDC45	5.E-36	54.40-
MMP16	3.E-23	49.40-
GTSE1	4.E-34	47.83-
PRR11	8.E-85	46.50-
TOP2A	4.E-85	46.18-
AURKB	6.E-45	45.99-
HIST1H3F	3.E-71	42.55-
MYB	1.E-65	41.97-
BIRC5	5.E-57	38.35-
RRM2	5.E-28	37.27-
SPDEF	1.E-28	36.37-
KIF18B	4.E-48	36.30-
CDCA3	2.E-42	35.91-
KIF4A	6.E-37	35.11-
HIST1H3C	8.E-57	34.00-
NCAPH	2.E-39	33.46-
TFF3	2.E-07	33.46-
KIF20A	5.E-57	33.43-
HIST1H1B	2.E-83	32.63-
ACKR3	5.E-21	32.61-

Gene	FDR step up	Fold change
ADCY1	2.E-97	32.48-
CLSTN2	4.E-75	31.99-
E2F1	6.E-50	31.55-
HJURP	2.E-47	31.50-
ASPM	2.E-67	30.85-
MCM10	2.E-49	30.36-
MALRD1	8.E-67	30.09-
KIF15	1.E-35	29.50-
SPC24	5.E-45	28.74-
CDK1	3.E-38	28.13-
TROAP	8.E-64	28.01-
HIST1H2AJ	3.E-25	27.89-
KCNJ3	4.E-52	27.75-
PLXNA4	3.E-45	27.60-
ANLN	4.E-58	26.96-
GFRA1	8.E-79	26.56-
TMEM64	3.E-73	26.54-
CENPF	6.E-78	26.11-
HIST1H4A	1.E-40	25.91-
ANXA6	1.E-11	25.87-
FOXM1	9.E-69	25.81-
FREM2	2.E-55	25.53-
IGFBP5	2.E-17	25.30-
DTL	5.E-27	24.99-
ZNF367	8.E-38	24.76-
KIF2C	6.E-44	24.64-
NCAPG	3.E-29	23.91-
GSTM3	2.E-81	23.79-
HIST1H3G	1.E-58	23.16-
ATP8A2	5.E-22	22.93-

Table S2: Comparison of TME-stimulated CSCs and non-stimulated cells: 60 top up-regulated genes and 60 top down-regulated genes

RNAseq analyses were performed on TME-stimulated CSCs and on non-stimulated cells (MCF-7 cells), as described in Figure 2. The Table demonstrates **(A)** 60 genes that were most up-regulated in TME-stimulated CSCs compared to non-stimulated cells and **(B)** 60 genes that were most down-regulated in TME-stimulated CSCs compared to non-stimulated cells.

A. Up-regulated genes

Gene	FDR step up	Fold change
LINC02015	2.E-57	134.81
SAMD9L	2.E-33	121.66
TCIM	4.E-30	100.03
BIRC3	2.E-58	95.64
IL6	3.E-17	72.32
CXCL8	4.E-36	63.76
GBP1	2.E-64	58.32
C1S	9.E-40	56.62
S100A9	2.E-41	55.40
TRIM22	2.E-52	55.05
CLDN1	9.E-14	41.22
TLR6	7.E-14	36.22
CALD1	1.E-08	35.73
ITGAM	1.E-36	31.74
CTSS	4.E-18	31.09
GBP4	2.E-27	29.70
DAPP1	4.E-32	26.98
C3	1.E-42	26.87
IFNB1	2.E-11	24.33
C1R	2.E-31	22.96
PRAL	2.E-06	21.63
SLC28A3	5.E-23	20.97
KRT17	2.E-22	19.88
KLHDC7B	3.E-59	19.47
IRAK2	1.E-37	19.15
IFIT2	1.E-42	17.91
C4orf54	2.E-22	16.66
IFI44L	1.E-15	15.78
IFI44	4.E-39	15.46
LAMC2	1.E-52	15.07

Gene	FDR step up	Fold change
ARHGEF38	1.E-32	14.60
RSAD2	2.E-36	14.35
CCL22	6.E-09	14.27
IFNL1	7.E-14	14.20
BTN3A3	1.E-22	14.01
C15orf48	7.E-20	13.80
TLR2	3.E-33	13.77
TNFAIP3	1.E-60	13.55
TMEM156	9.E-17	13.48
SOD2	8.E-63	13.16
ERAP2	3.E-24	12.90
DDX60L	5.E-39	12.29
AC006460.2	1.E-10	12.03
CASC15	3.E-30	11.93
IFIT3	3.E-32	11.27
TRAF1	5.E-26	10.99
ICAM1	4.E-44	10.75
MMP13	3.E-13	10.53
FGD6	6.E-47	10.25
MMP9	7.E-28	10.21
XAF1	2.E-29	10.12
NPHS1	7.E-12	9.70
STRA6	4.E-29	9.59
LCN2	7.E-12	9.55
PLS3	8.E-05	9.28
CCDC33	2.E-17	9.20
MX2	1.E-21	9.11
RTP4	2.E-08	8.73
SAMD9	5.E-39	8.71
BTN3A1	2.E-19	8.67

B. Down-regulated genes

Gene	FDR step up	Fold change
RAMP3	6.E-69	279.71-
KIF18B	8.E-51	54.08-
MT-RNR1	1.E-02	46.35-
AC138811.2	9.E-48	44.09-
BIRC5	2.E-59	42.69-
HIST1H3B	2.E-67	39.93-
MYBL2	1.E-20	37.68-
HIST1H3C	3.E-58	35.48-
PRR11	1.E-79	35.34-
HIST1H3F	2.E-67	35.26-
TROAP	4.E-68	34.75-
IQGAP3	2.E-76	34.01-
MKI67	2.E-84	33.95-
MT-RNR2	2.E-02	33.66-
AURKB	9.E-46	32.93-
HIST1H1B	2.E-83	32.71-
AC112777.1	8.E-32	31.93-
SPC24	4.E-47	31.81-
TOP2A	3.E-73	31.28-
CDC45	9.E-36	31.05-
KIF4A	5.E-37	30.96-
CLSTN2	2.E-72	28.84-
UHRF1	4.E-51	28.80-
APOD	5.E-14	28.73-
GTSE1	2.E-33	28.00-
ADCY1	2.E-84	25.31-
PCP4	2.E-23	25.14-
SPDEF	1.E-23	24.89-
GPR68	1.E-28	24.60-
KIF2C	2.E-44	24.01-

Gene	FDR step up	Fold change
HJURP	9.E-44	23.97-
KIF20A	2.E-51	23.21-
KCNJ3	1.E-49	22.98-
KIF15	3.E-34	22.58-
CDCA3	2.E-40	21.95-
CIT	1.E-77	21.69-
PLXNA4	1.E-40	20.99-
MMP16	5.E-19	20.38-
HIST1H3I	3.E-37	20.15-
FOXM1	1.E-61	19.85-
HIST1H2AL	2.E-35	19.80-
DTL	2.E-25	19.59-
HIST1H4A	3.E-36	19.27-
E2F1	2.E-42	19.13-
NCAPH	4.E-34	18.96-
RRM2	4.E-19	18.72-
PRRT1B	2.E-40	18.57-
KIF14	9.E-37	18.46-
TK1	6.E-44	18.04-
TICRR	3.E-44	18.02-
ZNF367	2.E-33	17.84-
MCM10	8.E-43	17.71-
ESPL1	5.E-67	17.64-
PCDH10	5.E-40	17.55-
TP73	2.E-29	17.53-
ASPM	6.E-53	17.02-
CDC20	5.E-27	16.98-
HIST1H3G	4.E-50	16.90-
CDK1	4.E-33	16.90-
PRC1	2.E-52	16.84-

Table S3: Comparison of TME-stimulated Non-CSCs and non-stimulated cells: 60 top up-regulated genes and 60 top down-regulated genes

RNAseq analyses were performed on TME-stimulated Non-CSCs and on non-stimulated cells (MCF-7 cells), as described in Figure 2. The Table demonstrates **(A)** 60 genes that were most up-regulated in TME-stimulated Non-CSCs compared to non-stimulated cells and **(B)** 60 genes that were most down-regulated in TME-stimulated Non-CSCs compared to non-stimulated cells.

A. Up-regulated genes

Gene	FDR step up	Fold change
ZFP91-CNTF	6.E-07	3.81
SMIM15-AS1	2.E-02	2.97
MKRN5P	2.E-02	2.71
LRRC4B	1.E-02	2.70
IL17REL	2.E-02	2.30
MUC3A	4.E-02	2.18
CYP2W1	3.E-02	2.05
CCDC33	1.E-02	2.05
AC069281.2	3.E-02	1.99
PYGM	4.E-02	1.88
LINC00511	2.E-02	1.88
PERM1	1.E-02	1.84
SLC7A5P2	3.E-02	1.74
C17orf82	2.E-02	1.69
STRA6	5.E-02	1.69
CD44	2.E-02	1.66
ADIRF-AS1	3.E-02	1.66
SSPO	5.E-02	1.64
NEAT1	2.E-02	1.56
RTEL1-TNFRSF6B	1.E-02	1.54
MYO15B	3.E-02	1.48

B. Down-regulated genes

Gene	FDR step up	Fold change
CALD1	1.E-02	7.61-
ACTG2	5.E-05	7.38-
MATN3	2.E-03	6.41-
CLDN1	5.E-03	6.20-
AL590004.3	3.E-07	5.37-
MID1	4.E-04	5.24-
KIF5C	1.E-07	5.18-
DIO2	3.E-05	5.08-
SNORA12	5.E-05	5.04-
COL12A1	6.E-05	4.93-
PLS3	4.E-02	4.87-
SNORA79B	4.E-04	4.77-
SNORD10	2.E-05	4.60-
TP63	6.E-07	4.56-
NT5E	8.E-07	4.44-
SNORA57	3.E-06	4.30-
BCL2	9.E-07	4.30-
ANXA6	4.E-02	4.29-
ITGB6	1.E-11	4.21-
TENM3	3.E-07	4.18-
CXCL8	2.E-08	4.15-
SNORA73B	6.E-09	4.07-
SNORA17B	2.E-03	4.04-
C14orf132	7.E-09	3.96-
TGFB2	1.E-12	3.94-
SCUBE2	3.E-03	3.75-
DCDC2	2.E-03	3.72-
SH3BGRL	3.E-07	3.71-
SNORA2C	1.E-04	3.69-
TUBA1A	1.E-07	3.68-

Gene	FDR step up	Fold change
LINC00052	1.E-02	3.67-
FSTL4	3.E-04	3.56-
GPC6	1.E-03	3.48-
SNORA73A	2.E-03	3.47-
RNU2-2P	1.E-03	3.46-
MSRB3	1.E-02	3.45-
LIN7A	2.E-06	3.40-
DAB2	3.E-02	3.34-
SCARNA22	6.E-03	3.32-
FREM2	3.E-06	3.32-
SNORD17	4.E-03	3.32-
SLC26A2	3.E-06	3.30-
SCARNA5	3.E-03	3.29-
MACC1	1.E-05	3.27-
SNORD3A	8.E-07	3.25-
AC079414.2	4.E-02	3.18-
AP005263.1	1.E-02	3.14-
ATP8A2	2.E-02	3.12-
SNORA49	6.E-04	3.12-
SEMA3D	2.E-05	3.07-
IGSF3	2.E-02	3.04-
RET	2.E-04	3.02-
AREG	3.E-05	2.99-
CLEC3A	3.E-03	2.97-
FAM234B	7.E-05	2.97-
DSC2	2.E-04	2.95-
SESN3	3.E-04	2.94-
FERMT1	2.E-02	2.93-
MIR205HG	4.E-03	2.92-
IFI44L	3.E-02	2.89-

Table S4: Comparison of TME-stimulated CSCs and TME-stimulated Non-CSCs cells: All up-regulated genes and 60 most down-regulated genes

RNAseq analyses were performed on TME-stimulated CSCs and TME-stimulated Non-CSCs (MCF-7 cells), as described in Figure 2. The Table demonstrates **(A)** All genes that were up-regulated in TME-stimulated CSCs compared to Non-CSCs cells and **(B)** 60 genes that were most down-regulated in TME-stimulated CSCs compared to Non-CSCs cells.

A. Up-regulated genes

	CSCs *vs.* Non-CSCs		CSCs *vs.* No Stimulation		Non-CSCs *vs.* No Stimulation	
Gene symbol	FDR	Fold Change	FDR	Fold Change	FDR	Fold Change
CXCL8	0.0000	4.1543-	0.0000	15.3476	0.0000	63.7580
IL6ST	0.0020	2.0894-	0.0003	1.9867	0.0000	4.1508
CCL22	0.7679	1.3518	0.0000	19.2920	0.0000	14.2719
IL6	0.1649	1.8546-	0.0000	38.9928	0.0000	72.3155
IL23A	0.9182	1.0827-	0.0001	3.6568	0.0000	3.9593
CLCF1	0.9586	1.0289	0.0072	1.6122	0.0133	1.5669
LIF	0.9900	1.0068	0.0000	5.5114	0.0000	5.4739
IFNB1	0.7545	1.2417-	0.0000	19.5904	0.0000	24.3257
CSF1	0.2461	1.4393-	0.0000	4.1829	0.0000	6.0202
IL15RA	0.8901	1.1024-	0.0222	1.9486	0.0091	2.1481
OSMR	0.0591	1.7145-	0.0000	2.2966	0.0000	3.9376
IL20RB	0.6164	1.3314	0.0009	2.7974	0.0220	2.1012
IFNGR1	0.0515	1.6672-	0.0000	2.6794	0.0000	4.4671
FAS	0.9895	1.0092	0.0000	3.7066	0.0000	3.6727
IL16	0.5181	1.3874	0.0278	1.8727	0.3392	1.3497
IL6R	0.4570	1.3330-	0.3930	1.2240	0.0292	1.6316
LIFR	0.5757	1.2357-	0.1515	1.3527	0.0123	1.6716

B. Down-regulated genes

	CSCs *vs.* Non-CSC		CSCs *vs.* No Stimulation		Non-CSCs *vs.* No Stimulation	
Gene symbol	FDR	Fold Change	FDR	Fold Change	FDR	Fold Change
CXCL12	0.0216	2.1259-	0.0000	6.5841-	0.0000	3.0970-
IL1R1	0.0451	2.3117-	0.0000	14.2121-	0.0000	6.1480-
TPO	0.5145	1.5279-	0.0000	8.6078-	0.0000	5.6338-
IL17D	0.7930	1.1685-	0.0002	2.5447-	0.0021	2.1778-
EPOR	0.9978	1.0026-	0.0005	2.2340-	0.0005	2.2281-
IL10RB	0.7128	1.1678-	0.0032	1.7640-	0.0374	1.5105-
IL17RC	0.7868	1.1219-	0.0000	2.3079-	0.0001	2.0571-
IL17RB	0.9173	1.0745-	0.0006	2.3322-	0.0019	2.1706-
LTBR	0.8357	1.0857	0.0285	1.4359-	0.0075	1.5589-
NGFR	0.8205	1.1872-	0.0088	2.2786-	0.0418	1.9193-
CCL28	0.2033	1.5519-	0.0049	1.8597-	0.4547	1.1983-
CXCR5	0.3576	1.3874-	0.0356	1.5627-	0.6206	1.1264-
IL13RA1	0.0592	1.6326-	0.0052	1.6630-	0.9349	1.0186-
PRLR	0.1683	1.4764-	0.0002	1.9672-	0.1399	1.3324-
IL17RA	0.7321	1.1495-	0.0136	1.5853-	0.0994	1.3791-
IL17RE	0.2919	1.6146-	0.0072	2.1041-	0.3808	1.3032-
IFNAR1	0.0154	1.8397-	0.1411	1.3314-	0.0992	1.3818

Table S6: Cytokines and chemokines that were differentially expressed in TME-stimulated CSCs, TME-stimulated Non-CSCs and non-stimulated cells
RNAseq analyses were performed on TME-stimulated CSCs, TME-stimulated Non-CSCs and on non-stimulated cells (MCF-7 cells), as described in Figure 2. The Table demonstrates all cytokines and chemokines that were detected in this analysis, sorted to: **(A)** Up-regulated genes in TME-stimulated CSCs, when compared to TME-stimulated Non-CSCs; in TME-stimulated CSCs, when compared to No stimulation; in TME-stimulated Non-CSCs, when compared to No stimulation. **(B)** Down-regulated genes in TME-stimulated CSCs, when compared to TME-stimulated Non-CSCs; in TME-stimulated CSCs, when compared to No stimulation; in TME-stimulated Non-CSCs, when compared to No stimulation. Differentially-expressed genes that passed the cutoff FC≥2 or FC≤−2, with pFDR<0.05 are marked in blue.
